# An Indocyanine Green‐Based Nanoprobe for *In Vivo* Detection of Cellular Senescence

**DOI:** 10.1002/anie.202404885

**Published:** 2024-05-16

**Authors:** Andrew G. Baker, Muhamad Hartono, Hui‐Ling Ou, Andrea Bistrović Popov, Emma L. Brown, James Joseph, Monika Golinska, Estela González‐Gualda, David Macias, Jianfeng Ge, Mary Denholm, Samir Morsli, Chandan Sanghera, Thomas R. Else, Heather F. Greer, Aude Vernet, Sarah E. Bohndiek, Daniel Muñoz‐Espín, Ljiljana Fruk

**Affiliations:** ^1^ Early Cancer institute Department of Oncology University of Cambridge Hills Road Cambridge CB2 0XZ UK; ^2^ Department of Chemical Engineering and Biotechnology University of Cambridge Philippa Fawcett Drive Cambridge CB3 0AS UK; ^3^ Department of Physics University of Cambridge JJ Thomson Avenue CB3 0HE United Kingdom; ^4^ Cancer Research UK Cambridge Institute Robinson Way Cambridge CB2 0RE UK; ^5^ School of Science and Engineering University of Dundee Dundee DD1 4HN Scotland UK; ^6^ Instituto de Biomedicina de Sevilla, IBIS/Hospital Universitario Virgen del Rocio Universidad de Sevilla Avda.Dr. Fedriani/> Sevilla 41009 Spain; ^7^ Yusuf Hamied Department of Chemistry University of Cambridge Lensfield Road Cambridge CB2 1EW UK

**Keywords:** Cellular senescence, cancer, endocytosis, ICG nanoprobe, photoacoustic tomography (PAT)

## Abstract

There is an urgent need to improve conventional cancer‐treatments by preventing detrimental side effects, cancer recurrence and metastases. Recent studies have shown that presence of senescent cells in tissues treated with chemo‐ or radiotherapy can be used to predict the effectiveness of cancer treatment. However, although the accumulation of senescent cells is one of the hallmarks of cancer, surprisingly little progress has been made in development of strategies for their detection in vivo. To address a lack of detection tools, we developed a biocompatible, injectable organic nanoprobe (NanoJagg), which is selectively taken up by senescent cells and accumulates in the lysosomes. The NanoJagg probe is obtained by self‐assembly of indocyanine green (ICG) dimers using a scalable manufacturing process and characterized by a unique spectral signature suitable for both photoacoustic tomography (PAT) and fluorescence imaging. In vitro, ex vivo and in vivo studies all indicate that NanoJaggs are a clinically translatable probe for detection of senescence and their PAT signal makes them suitable for longitudinal monitoring of the senescence burden in solid tumors after chemotherapy or radiotherapy.

## Graphical abstract

Sound of Senescence: A nanostructured organic probe, NanoJaggs, can be used as photoacoustic contrast agent *for* in vivo imaging of the senescent cell burden in post‐chemotherapy tumors. Made of indocyanine green (ICG) dimers, 30 nm nanoparticles exploit an active endocytosis mechanism and increase the number of lysosomes to specifically light up senescent cells implicated in cancer initiation and progression.

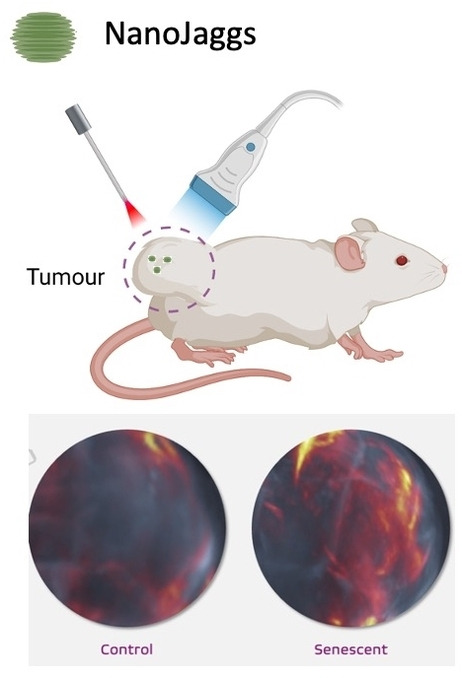

## Introduction

Cellular senescence is a response to irreparable damage and stress resulting in stable cell cycle arrest, which can prevent the expansion of altered, potentially pathological, cells. This triggers the secretion of a complex mixture of inflammatory and tissue remodeling factors (**s**enescence‐**a**ssociated **s**ecretory **p**henotype, known as SASP) to aid the repair of the surrounding tissue. Under normal conditions, the SASP can recruit inflammatory cells to drive senescent cell clearance ultimately executed by phagocytic cells.[^1^, ^2^] However, persistent damage and stress can result in dysregulation of immunosurveillance and the accumulation of senescent cells in tissues. Such an accumulation promotes the onset and progression of multiple age‐related and chronic disorders including cardiovascular diseases, fibrosis, neurological disorders, obesity, inflammatory syndromes and cancer.[^1^, ^3^] The important role of senescence in the development of variety of pathologies has been acknowledged by its inclusion within the hallmarks of both ageing and cancer.[^4^, ^5^]

Although senescence can be a context‐dependent antagonistic response, studies have shown that the removal of senescent cells by pharmacogenetic approaches can significantly promote (by ~30 %) the healthspan and lifespan in progeroid and naturally‐aged mice.[^6^, ^7^] These landmark studies accelerated development of therapeutic approaches such as senolytic compounds to eradicate senescent cells, and senomorphics and senostatics for regulation of the inflammatory SASP.[^3^, ^8^, ^9^, ^10^, ^11^, ^12^] In case of cancer, combination of both pro‐ and anti‐senescence treatments referred to as the *“one‐two punch”* approach has shown particularly promising results and has been highlighted as a promising future strategy within precision oncology.[^11^, ^12^, ^13^, ^14^]

Considering the important role of cellular senescence in disease development as well as the recent advances in the field of senotherapies, surprisingly little progress has been made in the design of contrast agents and tools for in vivo detection of the senescence burden. This can partially be explained by the lack of an universal biomarker of senescence, thus, senescence detection often requires a multistep or algorithmic approaches based on an array of biomarkers.[^15^, ^16^] Most commonly employed are the expression of tumor suppressor p53 and cell cycle inhibitor proteins p16 and/or p21 to ascertain the cell cycle arrest; and senescence‐associated β‐galactosidase (SA‐β‐gal) phenotype, a colorimetric reaction based on increased lysosomal β‐galactosidase enzymatic activity in senescent cells. Although widely used, enhanced SA‐β‐gal activity is not a distinctive feature of only senescent cells, but it is also present in other cell types such as osteoclasts, neurons and macrophages.^[17]^ Despite limited specificity, enhanced activity of lysosomal β‐galactosidase was employed in the design of small molecule fluorescent senoprobes.[^18^, ^19^, ^20^] However, fluorescence‐based strategies are not well suited for clinical applications because of their poor penetration depth,^[10]^ limiting the imaging of internal organs which requires a penetration depth of up to several centimeters.[^21^, ^22^] To address this limitation, two positron emission tomography (PET) probes based on SA‐β‐gal activity have recently been reported for detection of senescence.^[23]^ Although PET is widely employed as a clinical imaging strategy, it requires the use of radioactive traces, sophisticated imaging systems, as well as specialized infrastructure. In addition to PET, magnetic resonance imaging (MRI), and computed tomography (CT), photoacoustic tomography (PAT) has recently emerged as a powerful in vivo imaging technique^[24]^ with significant potential for early detection of cancer.[^25^, ^26^, ^27^, ^28^]

PAT employs a pulsed laser to excite the appropriate contrast agent, resulting in conversion of absorbed light into acoustic energy. As acoustic waves scatter to a lesser extent than light, PAT images can be constructed from larger penetration depth compared to light microscopy techniques. In contrast to CT and PET, PAT does not require ionizing radiation, the acquisition times are shorter, and the instruments more affordable and easier to operate.^[25]^ Importantly, by acquiring images at several wavelengths, PAT allows for multiplexed imaging, resulting in data sets that reveal the distribution of several chromophores.^[26]^

Both endogenous molecules such as hemoglobin, and exogenous near‐infrared (NIR) absorbing dyes can be used as PAT contrast agents.^[29]^ Broadly speaking, an ideal PAT contrast agent should have a high molar extinction coefficient and exhibit a distinct, well‐defined absorption spectrum to maximize the light absorption and facilitate differentiation from other chromophores.^[29]^ In addition, it should be photostable while maintaining a low quantum yield to ensure efficient conversion of light energy into heat.^[30]^ These prerequisites make nano‐based probes exceptionally well‐suited, given their tunable absorption spectra, high extinction coefficients and a large surface area that allows for further (bio)functionalization with stabilizing and targeting agents.[^31^, ^32^, ^33^]

Herewith, we describe design and validation of a novel PAT nanoprobe for in vivo detection of senescent cells. The nanoprobe was prepared by exploiting self‐assembly of indocyanine green (ICG) dimers into J‐aggregate structures under mild and scalable conditions. J‐aggregates, initially described nearly 100 years ago, are supramolecular assemblies of organic dyes such as cyanines, porphyrins and perylene bisimides, characterized by unique optical and photophysical properties.[^34^, ^35^] A number of nanostructured J‐aggregates, ranging in sizes from 90 to 130 nm, have been prepared to date, mainly for tumor imaging and photothermal therapy.[^36^, ^37^, ^38^, ^39^, ^40^] Herewith, we describe nanosized J‐aggregate probe (NanoJagg) characterized by high purity, remarkable physiochemical properties, long‐term stability under conventional storage conditions and biocompatibility. NanoJaggs can be manufactured in a scalable and reproducible way and employed for detection of chemotherapy‐induced senescence *in vitro*, *ex vivo* and *in vivo* using both fluorescence and PAT imaging.

## Results and Discussion

### Synthesis and Characterization of NanoJagg Probes

**Figure 1:**
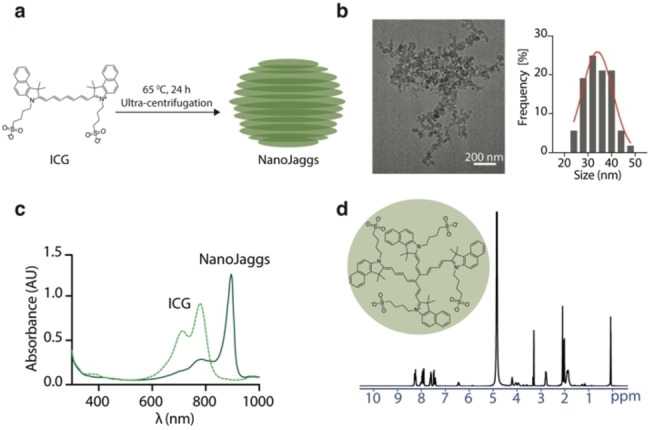
Synthesis and characterization of NanoJagg probe. (a) NanoJaggs are prepared by stirring of an aqueous solution of indocyanine dye (ICG) at 65 °C for 24 h. (b) Cryo‐EM of the resulting spherical nanoparticle indicated an average size of 34±4.8 nm. (c) Characteristic red absorbance shift from 780 (ICG) to 895 nm is observed upon NanoJagg formation. (d) ^1^H NMR spectra indicate the presence of ICG dimers as the main NanoJagg building blocks.

Pure NanoJaggs were prepared by stirring an aqueous solution of indocyanine green (ICG) dye at 65 °C for 24 h (Figure 1a), and subsequently purified by dialysis and ultra‐centrifugation (Figure 1b). The centrifugation step is crucial, as previous studies that omitted this purification step obtained a complex mixture of J‐aggregates and side products (Figure 1S).[^36^, ^37^, ^38^, ^39^] This raises concerns about the reliability of the conclusions drawn from these earlier studies, as J‐aggregates and their individual building blocks likely have different impact on cells. Assembly of NanoJaggs was monitored by measuring the absorbance of J‐aggregates at λ_max_=895 nm and ICG at λ_max_=780 nm (Figure 1c). Both NMR and LC–MS/MS analyses were performed to better understand the structural composition of the pellet and supernatant obtained after centrifugation (Figure S2 to S4). A single peak with 751 m/z was observed in the chromatogram of the pellet (Figure S3b), while the chromatogram of the supernatant contained multiple peaks, some of which have been identified as ICG degradation products (Figure S4b to d).

The disappearance of the ^1^H NMR peak at 6.60 ppm corresponding to the hydrogen in the carbonyl chain of ICG indicates the formation of the bridging bond between two ICG molecules within NanoJagg structure (Figure 1d). This was in line with previous studies reporting formation of ICG dimers.[^41^, ^42^] The ^1^H NMR study of the supernatant confirmed the presence of multiple compounds (Figure S4), which amounted to 54 % of the total yield by mass.

Unlike previously reported J‐aggregate nanostructures, NanoJaggs were chemically pure with 97 % dimer composition as demonstrated by HPLC (Figure S5). While dimerization of ICG has been noted when J‐aggregates formed in thin films of nanoemulsions,^[43]^ to the best of our knowledge, this is the first J‐aggregate nanoparticle reported that is entirely composed of dimeric ICG.

To gain a better understanding of the size distribution and shape of the obtained NanoJaggs, cryo‐electron microscopy (cryo EM) imaging was performed showing semi‐spherical NPs with an average diameter of 34±4.8 nm (Figure 1b and Figure S6a). Further, energy dispersive X‐ray (EDX) analysis demonstrated the presence of sulfur stemming from the sulfonate group on the ICG building blocks (Figure S6b).

The absorbance spectrum of NanoJaggs displays a red shift in comparison to ICG (λ_max_=895 nm and λ_max_=780, respectively Figure 1c), while the fluorescence spectra reveal quenching of ICG dimers within the aggregates (λ_em_=810 nm) (Figure S6c). Such quenching phenomenon is advantageous for photoacoustic imaging as it results in a higher proportion of absorbed energy being converted into acoustic energy, thus enhancing the PAT signature.^[44]^ In fact, when normalized to the same absorbance maximum, NanoJaggs exhibit a remarkable three‐fold enhancement in the overall generated photoacoustic signal (Figure S6d), which is in line with previous reports on photoacoustic properties of J‐aggregates.^[45]^ In addition, the extinction coefficient was calculated to be 1.92±0.07×10^9^ M^−1^ cm^−1^ (Figure S6e), which resembles that of metallic plasmonic nanoparticles,[^29^, ^46^] even though NanoJaggs are composed entirely of organic molecules.

Interestingly, when we compared NanoJaggs to ICG dimers in Dulbecco's Modified Eagle medium (DMEM) with 10 % fetal bovine serum (FBS) used for maintaining cell cultures, we observed that dimers rapidly assemble into NanoJaggs (Figure S7a). This assembly was temperature‐dependent and inhibited at low temperature (Figure S7 b and c). There were also no discernible changes in the absorbance spectra of NanoJaggs when assessed in different biologically relevant media (PBS buffer at pH 7.4, DMEM, DMEM with 10 % FBS and FBS only) over a span of 7 days indicating their remarkable colloidal stability (Figure S8). Remarkably, these nanoparticles can be stored for more than a year as lyophilized powder, or for at least 8 months as aqueous suspension, which significantly enhanced their potential for clinical use.

### Accumulation of NanoJaggs in Senescent Cells

Previously it was shown that ICG dye undergoes efficient uptake into the lysosomes of cancer cells lines.[^47^, ^48^] Therefore, we hypothesized that ICG‐containing NanoJaggs would leverage the benefits of ICG improved lysosomal uptake. Compared to cancer cells and normal (differentiated) cells, senescent cells are characterized by an enlarged lysosomal compartment, evident in both an increased size and a greater quantity of lysosomes.[^49^, ^50^, ^51^, ^52^] Consequently, significantly more NanoJagg probes would be expected to accumulate in senescent cells. To test this hypothesis, we first performed cell uptake and colocalization studies in human lung adenocarcinoma (A549) and melanoma (SK‐MEL‐103) cancer cell lines demonstrating that NanoJaggs, similarly to ICG, indeed target the lysosomal compartment (Figure S9).

**Figure 2:**
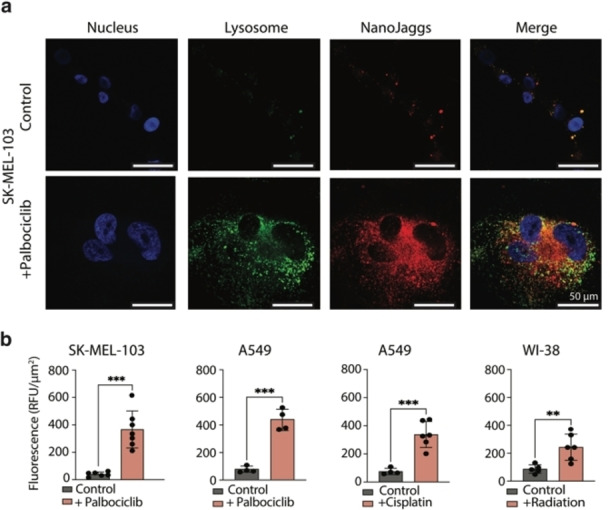
Selectivity and accumulation of NanoJaggs in senescent cells *in vitro*. (a) Confocal images of non‐senescent and senescent SK‐MEL‐103 cells induced by Palbociclib and treated with 50 μg/mL NanoJaggs. (b) Confocal quantification of relative fluorescent units (RFU)/μm^2^ of the NanoJagg signal in the different cell lines. Data represent as mean±SD (*n*=3 biological replicates), and a Two tailed t‐test was used to calculate the significance (*p<.05, **p<.01, ***p<.001). All examples used show a significant increase in NanoJagg uptake compared to control cells and adjusted for cell area.

Next, we wanted to explore the specificity of NanoJaggs towards senescent cells. Given that senescence is a heterogeneous response that depends on the trigger, cellular type and the context, various models of senescence were employed in this study. Specifically, senescence was induced in SK‐MEL‐103 and A549 cancer cells by treatment with chemotherapeutic Palbociclib, which selectively inhibits cyclin dependent kinases 4 and 6 (CDK4/6). In addition, DNA intercalating chemotherapy agent Cisplatin was employed in A549 cancer cells, and DNA‐damaging radiation was applied to human WI‐38 fibroblasts to serve as radiotherapy‐induced senescence model. The implementation of senescence status in these models was evaluated using established protocols including increased SA‐β‐gal activity, proliferation assays and enhanced expression of p21 cell cycle inhibitor. In addition, decrease in expression levels of phosphorylated retinoblastoma (Rb) protein, which is a marker of an active cell cycle progression, was determined by western blotting (Figure S10). Once senescence models were successfully established, confocal microscopy was used to image the accumulation of NanoJaggs in cells showing a substantial increase in all senescent cell models compared to their non‐senescent counterparts (Figure 2a and b; Movie 1 and Movie 2). It should be noted that relative fluorescence units were normalized by the total cell area (μm^2^) to account for aberrant morphology and enlarged size of senescent cells, thus, ensuring that the obtained average signal is comparable across different cell types.

The largest NanoJagg uptake was observed in senescent SK‐MEL‐103 and A549 cells (9.4× and 5.2×, respectively, when compared to their non‐senescent cancer cell counterparts), while radiation‐induced senescent WI‐38 fibroblasts showed only 2.7× higher fluorescent signal than the corresponding non‐irradiated cells (Figure 2b). These results were additionally confirmed by flow cytometry studies (Figure S11). While senescent cells are known to have increased autofluorescence,^[53]^ we have not observed any interference stemming from autofluorescence within NanoJagg's excitation and emission range (Figure S12 a and b).

Unlike for cancer cells, the accumulation of NanoJaggs in senescent cells was found to be dose dependent and resulted in elevated signal level, even at the lowest concentration tested (0.4 mg/mL) (Figure S13). This was in contrast to ICG dye, which showed significant uptake in both the control and senescent cells (Figure S14). We also evaluated the uptake of ICG dimers, however their rapid assembly into J‐aggregates resulted in the similar uptake pattern as observed for NanoJaggs (Figure S8 and S15).

Colocalization studies showed that NanoJaggs are largely accumulated in lysosomes situated in the perinuclear space (Figure 2b and Figure S16 and S17) while no noticeable accumulation was observed in mitochondria (Figure S16 and S18) or endoplasmic reticulum (Figure S16 and S19).

Additionally, biocompatibility of NanoJaggs was assessed by CellTiter‐Blue viability assays in both senescent and non‐senescent human lung adenocarcinoma (A549) and melanoma (SK‐MEL‐103) cell lines treated with wide range of NanoJagg concentrations (0.1–100 μg/mL). As it can be seen in supplementary Figure S20, no significant toxicity was observed within this range confirming that NanoJaggs are not toxic and can be safely used as a versatile senoprobe to detect multiple senescent phenotypes by fluorescence microscopy.

In addition to fluorescence microscopy, we also showed that conventional optical or light microscopy can be used to monitor selective accumulation of NanoJaggs in senescent cells (Figure S21). This capability renders NanoJaggs comparable to commercially used SA‐β‐galactosidase activity kits in terms of selectivity, but with the advantage of being applicable to live cells due to absence of toxicity.

Finally, NanoJagg detection potential was validated using two additional senescent cell models employing non‐cancerous cell lines. Senescent human umbilical vein endothelial cells (HUVECs), a primary endothelial cell line, were obtained after Palbociclib treatment, while mouse embryonic fibroblasts (MEFs) at high passage number of replicative cycles were employed as a model of age‐related replicative senescence. Both the conventional SA‐β‐gal and our novel NanoJagg staining were performed using these cell lines. Palbociclib‐treated HUVECs showed an increased number of SA‐β‐gal positive cells and a significant increase in the uptake of NanoJaggs (Figure S22). The same was observed with MEFs, which showed increased number of SA‐β‐gal positive cells at replicative passage 9, at which point a significant increase in NanoJagg uptake was also observed (Figure S23). This result indicates that besides being used as probes for chemo‐ and radiotherapy induced senescence, NanoJaggs can also be employed for detection of age‐related senescence.

### Mechanism of NanoJagg Uptake in Senescent Cells

**Figure 3:**
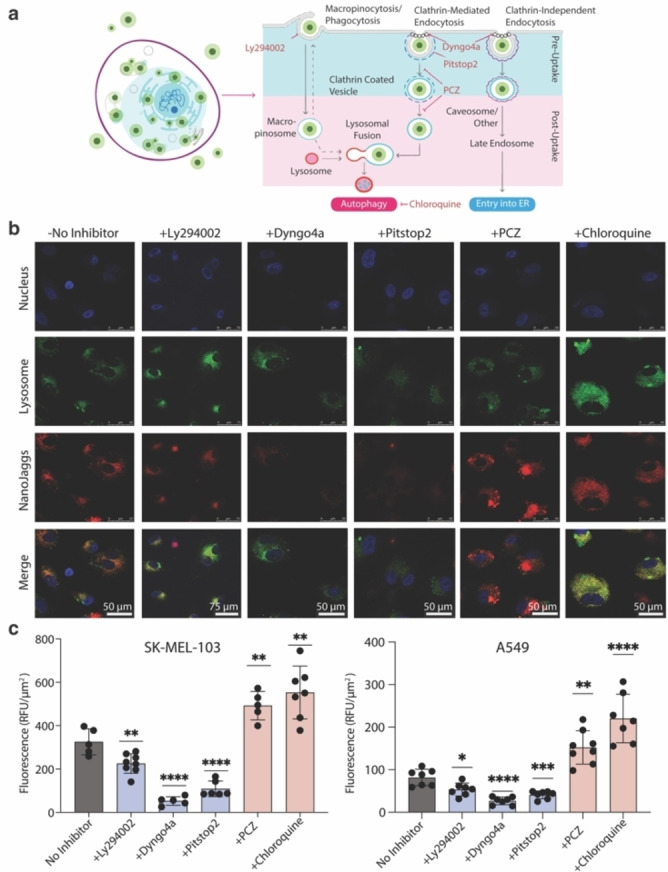
Mechanism of NanoJagg uptake in senescent cells. (a) Schematic representation of endocytosis in senescent cells and the inhibitors used in this study. Three pre‐uptake inhibitors were used (Pitstop 2, Dyngo4a and Ly294002), as well as two post‐uptake inhibitors (PCZ and Chloroquine). (b) Confocal images of NanoJagg uptake in senescent SK‐MEL‐103 cells treated with pre‐ and post‐uptake inhibitors of endocytosis. Lysosomes are stained with Lysotracker green and nuclei with Hoechst (blue). (c) Confocal quantification of relative fluorescent units (RFU/μm^2^) of SK‐MEL‐103 and A549 cells. Control cells not treated with inhibitors are shown in black. Pitstop 2 and Dyngo4a both result in a significant inhibition of NanoJagg uptake in both cell lines (p<0.0001), while Ly294002 caused lower but still significant reduction (p<0.01 for SK‐MEL‐103 and p<0.05 for A549). Both post‐uptake inhibitors result in an increase of NanoJagg signal: prochlorperazine's increase p<0.01 for SK‐MEL‐103, and p<0.05 for A549; Chloroquine causes p<0.01 for SK‐MEL‐103 and p<0.0001 for A549. Data represent mean±SD (*n*=3 biological replicates) and a Two‐tailed t‐test was used to calculate the significance (*p<0.05, **p<0.01, ***p<0.001, and ****p<0.0001).

After we observed a significant increase in NanoJaggs accumulation in various senescence models, we wanted to gain more insights into the underlying mechanism responsible for cellular uptake. Generally, active endocytosis pathways can be grouped into macropinocytosis (or phagocytosis), pinocytosis, receptor‐mediated endocytosis (also known as clathrin‐mediated endocytosis), and receptor‐independent endocytosis (involving cholesterol‐binding protein caveolin plasma membrane buds).^[54]^ As recent studies have identified altered endocytosis pathways in senescence,^[55]^ we set out to explore NanoJagg uptake in presence of pre‐ and post‐cellular uptake inhibitors (Figure 3a). Pre‐uptake endocytosis inhibitors either block cell membrane‐based pathways such as macropinocytosis (phophatidylinostitol‐3‐kinase inhibitor Ly294002) and clathrin‐coated pit formation (Pitstop 2), or inhibit the formation of dynamin protein (Dyngo4a) involved in clathrin‐ and caveolin‐ mediated endocytosis (structures of these inhibitors are shown in Figure S24).[^56^, ^57^, ^58^, ^59^] Post‐uptake inhibitors interfere with processes within the cell such as later stage of clathrin‐coated vesicle fission from the membrane (prochlorperazine, PCZ)[^56^, ^60^] and autophagy (Chloroquine).^[61]^

Significant decrease in NanoJagg uptake (p<0.0001) was observed when senescent SK‐MEL‐103 and A549 cancer cells were treated with inhibitors of clathrin‐mediated endocytosis (Pitstop 2 and Dyngo4a) (Figure 3b and c, Figure S25 and S26), with smaller changes observed upon administration of macropinocytosis inhibitor Ly294002 (p<0.01) (Figure 3b and c, Figure S25 and S26).

In contrast, non‐senescent, control SK‐MEL‐103 and A549 cells treated with pre‐uptake endocytosis inhibitors showed no significant differences in intracellular accumulation of NanoJaggs (Figure S27 and S28) indicating that increased endocytic uptake is a senescence‐dependent effect.

This aligns with previous studies that have shown an up‐regulation of endo‐lysosomal machinery as well as the activation of macropinocytosis (as a survival mechanism through nutrient scavenging) and phagocytosis (employed as a form of cell cannibalism) in senescent cells.[^62^, ^63^, ^64^, ^65^, ^66^] Indeed, due to their capacity to engulf other cells and synthesized materials,[^62^, ^63^, ^67^] senescent cells have often been compared to macrophages.^[67]^

Contrary to pre‐uptake endocytosis inhibitors, post‐uptake inhibitors PCZ and Chloroquine, resulted in increased NanoJagg fluorescence in both senescent and non‐senescent cell lines (Figure 3b and c, Figure S25 to S29). In addition, significantly lower co‐localization with lysosomes was observed after PCZ treatment, which could be attributed to PCZ‐mediated disruption of the fusion between clathrin‐coated vesicles and lysosomes.^[56]^ Chloroquine, on the other hand, is an inhibitor of autophagy that induces lysosome dilation and affects various processes downstream of lysosome formation, thus not impacting the colocalization (Figure S25 to S29).^[68]^

Together, these findings suggest that NanoJaggs are internalized through an active mechanism, likely involving both clathrin‐mediated endocytosis and macropinocytosis. While this phenomenon has been previously noted within the context of cell engulfment,^[62]^ its application in development of detection strategies or for improved drug delivery remains largely unexplored. Given that ICG has been observed to bind to the cancer cell membrane and subsequently undergo endocytosis,^[47]^ it is tempting to hypothesize that NanoJaggs could potentially recognize specific molecules on the senescent surfaceome thus enabling selective targeting. The identification of the senescent surfaceome represents an emerging area with significant potential for targeting senescent cells, and it warrants further investigation. Future studies will delve deeper into the mechanism of uptake to gain a more comprehensive understanding.

### Ex Vivo and In Vivo Assessment of Senescence in Chemotherapy‐Induced Xenograft Using NanoJaggs

**Figure 4:**
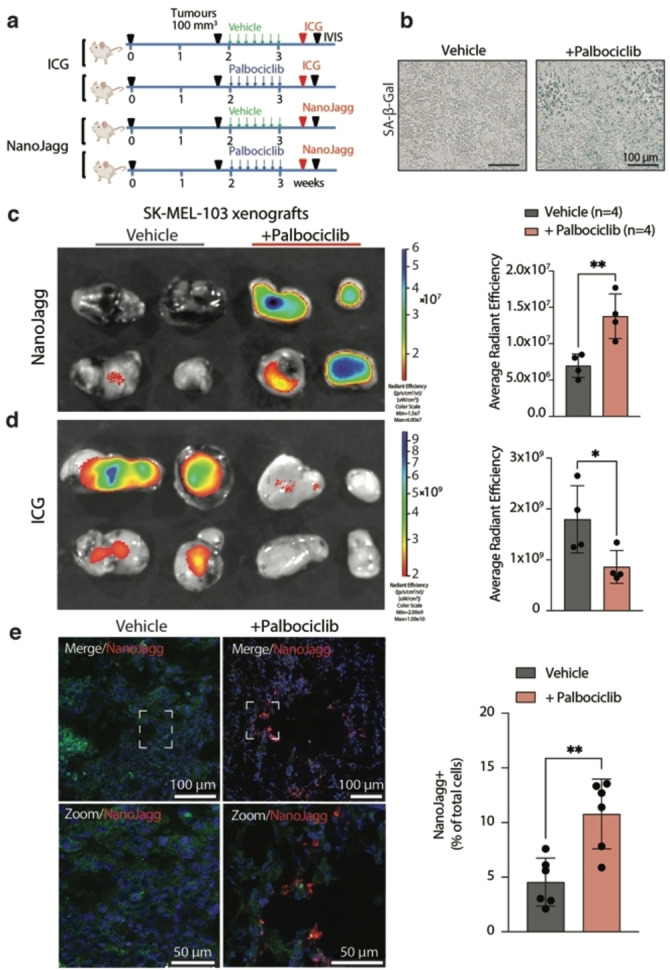
Fluorescence detection of NanoJaggs *ex vivo*. (a) Schematic overview of the *in vivo* experiment. The uptake of both ICG and the NanoJaggs were compared in Palbociclib‐treated tumors *vs* untreated tumors. (b) SA‐β‐ gal staining of untreated and Palbociclib‐treated tumors. (c) IVIS fluorescence images of dissected control (*n*=4) and senescent tumors (*n*=4) after treatment with NanoJaggs (left). Quantification of the average radiant efficiency comparing NanoJagg fluorescent signal (p=0.0077) is shown on the right. (d) IVIS images of dissected control and senescent tumors after treatment with ICG for 6 h (left). Quantification of the average radiant efficiency comparing ICG fluorescent signal (p=0.0438) is shown on the right. (e) Confocal images and quantification of NanoJagg‐positive cells comparing the untreated to Palbociclib treated tumors (*n*=4). Data represent mean±SD, and a Two‐tailed t‐test was used to calculate the significance. (*p<0.05, **p<0.01, ***p<0.001).

To assess the effectiveness of NanoJaggs for selective uptake into senescent cells, we utilized immunocompromised mice bearing tumor xenografts established using SK‐MEL‐103 melanoma cells treated with senescence‐inducing chemotherapy.^[13]^ After the formation of tumors, mice were subjected to daily treatment with Palbociclib over 7 days (Figure 4a). This regimen led to high levels of intertumoral senescence as indicated by an increase in SA‐β‐gal activity (Figure 4b), the absence of the proliferative marker Ki‐67, and a decrease in phosphorylated Rb protein levels.^[13]^ A single injection of vehicle (DMEM, phenol red‐free), NanoJaggs (200 μL at 1 mg/mL) or an equivalent amount of ICG dye were administered to the mice, and the fluorescent levels analyzed after 6 h by IVIS fluorescence imaging (Figure 4a). Significant levels of NanoJaggs fluorescence were detected in senescent tumors treated with Palbociclib (*n*=4) but not in control (proliferative) tumors (*n*=4) (p=0.0077) (Figure 4c). In contrast, ICG fluorescence was predominantly observed (p=0.0438) in non‐senescent tumors (*n*=4) (Figure 4d), which aligns with the previously reported tendency of ICG dye to accumulate in aggressive solid tumors.[^48^, ^69^]

Negligible background fluorescence signal in vehicle‐treated mice control (Figure S30a) confirmed that observed fluorescence can be predominantly attributed either to NanoJaggs or ICG within the tumors. Moreover, subsequent ex vivo biodistribution studies revealed that NanoJaggs fluorescence was hardly detectable 6 h after administration in organs of the reticuloendothelial system such as the liver and spleen, in both untreated and Palbociclib‐treated mice. In both mice groups, a considerable fluorescence signal was observed in the kidneys, suggesting that renal clearance is a preferential route of elimination (Figure S30b).

Confocal imaging of tissue slices was conducted to provide additional validation for the accumulation of NanoJaggs in the senescent tumors (Figure 4e, p=0.0028).

To further assess the effectiveness of NanoJaggs in targeting senescent cells, we also utilized immunocompromised mice bearing tumor xenografts established using A549 adenocarcinoma cells treated with cisplatin. After the formation of tumors, mice were subjected to intraperitoneal cisplatin or vehicle injections three times a week for 14 days (Figure S31a). This regimen led to high levels of intratumoral senescence as indicated by an increase in SA‐β‐gal activity and a reduced tumor growth rate (Figure S31b and e). Upon NanoJaggs administration via the tail vein injection, the signal‐to‐background ratio was significantly increased in cisplatin‐treated tumors (*n*=6) when compared to the vehicle control (*n*=6) (p<0.05.). In addition, the signal‐to‐background fluorescent ratio significantly increased (p<0.001) when cisplatin‐treated mice were compared pre‐ and post‐NanoJagg administration. This longitudinal monitoring clearly demonstrates that the signal increase stemmed solely from the accumulation of the nanoprobe within chemotherapy‐induced senescent tumors.

### NanoJaggs as Contrast Agents for Photoacoustic (PA) Imaging of Senescent Cell Burden In Vivo

Following the encouraging results obtained from fluorescence imaging, a subsequent study was conducted to explore the potential of NanoJaggs to be used as photoacoustic (PA) contrast agents. PA is an emerging imaging technique with substantial potential for translational applications in cancer and other diseases, as it enhances the imaging depth, all the while maintaining spatial and temporal resolution.[^25^, ^28^, ^29^, ^70^] Two infrared (IR) dyes, ICG and cetuximab‐800CW, are currently employed as PAT agents in clinical studies for lymph node mapping in melanoma (NCT05467137) and cervical cancer (NCT03923881), respectively. Moreover, several PAT nanoparticle contrast agents, both metal nanoparticles and organic structures, have been reported and successfully validated in preclinical studies.^[29]^

**Figure 5:**
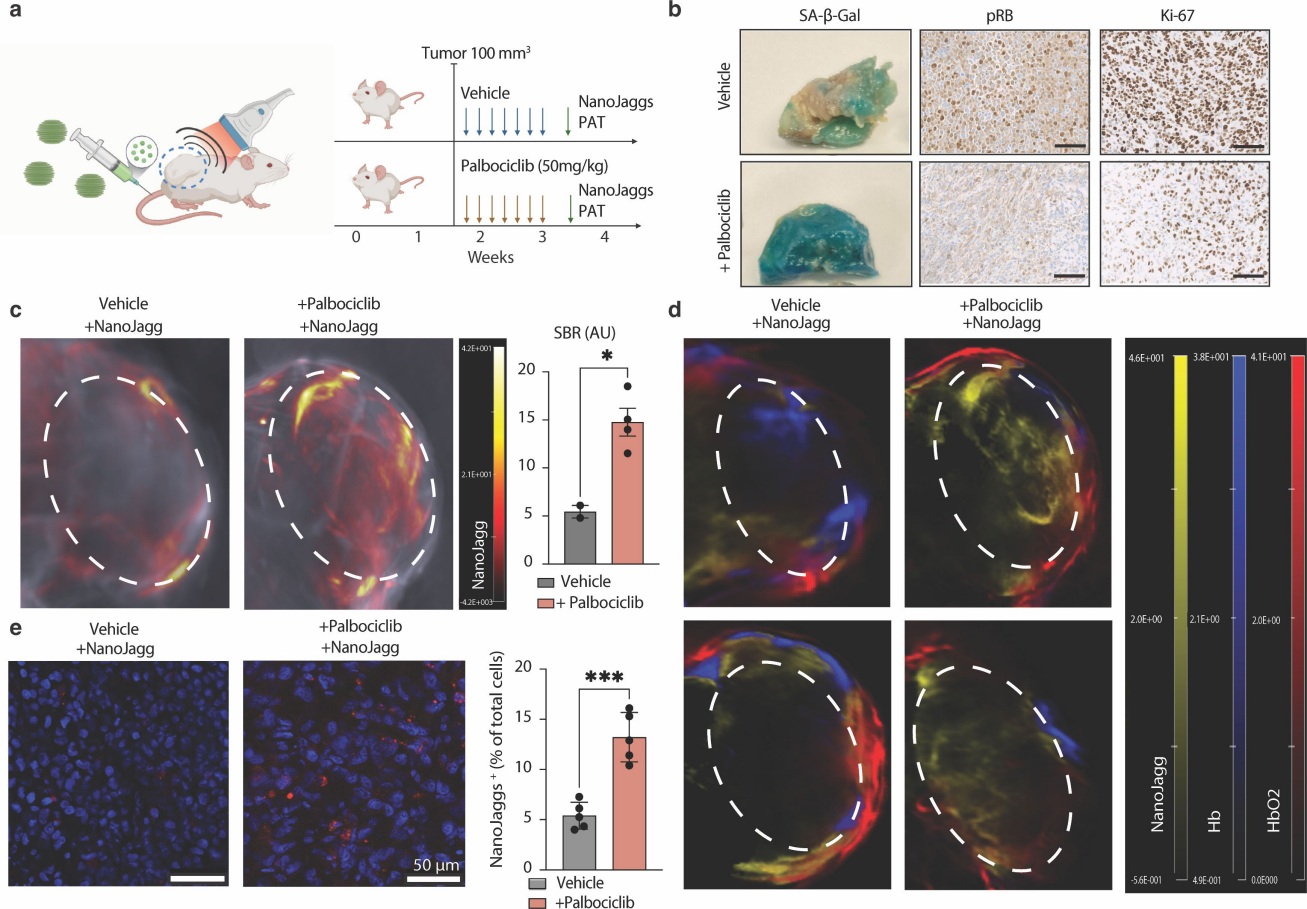
Photoacoustic imaging of senescent cells in live mice employing NanoJagg probe. (a) Experimental design using two groups of xenograft mice transplanted with SK‐MEL‐103 cells: treated with Palbociclib to induce senescence, and a control vehicle. NanoJaggs were injected into both groups through tail vein. (b) Assessment of senescence in tumors post‐*in vivo* imaging. Palbociclib‐treated, senescent tumors show strong SA‐β‐Gal activity and decrease in nuclear pRB and Ki‐67 levels compared to control tumors. Scale bars=100 mm. (c) Representative axial images (top) of control (vehicle+NanoJaggs, *n*=2) and senescent tumors (Pablociclib+NanoJaggs, *n*=4) treated with NanoJaggs. Significant difference can be observed in signal to background ratio (SBR, bottom) of PAT signal in control and senescent tumor. (d) PAT Images obtained by spectral unmixing of NanoJagg (yellow), Hb (blue) and HbO_2_ (red). Bottom image represents scale bar for both (c) and (d). (e) Representative confocal images of biopsied tissue sections post‐*in vivo* PAT imaging. In both cases multiple tumors were used, as well as imaged in different parts of each slice. Quantification of NanoJagg‐positive cells indicates significant differences between senescent and control mice. Data represent mean±SD, and a Two‐tailed t‐test was used to calculate the significance (*p<0.05, **p<0.01, ***p<0.001).

To evaluate NanoJaggs potential for PAT, we employed immunocompromised female nude mice bearing SK‐MEL‐103 melanoma tumor xenografts, divided into Palbociclib‐ or vehicle‐treated groups. NanoJaggs or ICG were administered via tail vein injection and PAT imaging carried out at intervals of 6 and 24 h post‐injection (Figure 5a). Induction of senescence within tumors was confirmed after in vivo imaging by SA‐β‐gal activity assays and immunohistochemistry (IHC) staining of pRB and Ki‐67 (Figure S32a). As expected, the Palbociclib‐treated group showed an increased lysosomal SA‐β‐gal activity and a decreased nuclear staining of pRB and Ki‐67, indicating a reduced proliferative capacity and the implementation of the senescent program (Figure 5b). In addition, tumors treated with Palbociclib showed a marked reduction in proliferation rates, thereby confirming the onset of cell cycle arrest characteristic of senescence (Figure S32c to e).

As it can be observed in Figure 5c, the NanoJaggs signal was prominently distributed throughout the senescent tumor of Palbociclib‐treated mice. In contrast, in the control (non‐senescent, treated with vehicle) proliferative tumors, the signal was concentrated at the tumor edges. The signal‐to‐background ratio (SBR) was determined by dividing the signal from the tumor region of interest (ROI) by the background signal obtained through spectral unmixing of the NanoJagg PAT spectrum (Figure 5c). This SBR was computed for every axial plane of both control and Palbociclib‐treated tumors, providing a representative NanoJagg signal across the entire tumor volume. A notably higher SBR was observed in the senescent, Palbociclib‐treated tumors (*n*=4), in comparison to the proliferative tumor counterparts (*n*=2) (p=0.0135). This observation was corroborated by comparable values obtained through quantification based on contrast‐to‐noise ratios (Figure S33a).

One of the advantages of PAT imaging lies in its ability to acquire and analyze multiple data streams, including parameters such as blood oxygenation and the co‐colocalization of blood vessels using distinct signals from de‐oxygenated (Hb) and oxygenated (HbO_2_) hemoglobin. Linear spectral unmixing applied to hemoglobin (Hb), deoxyhemoglobin (HbO_2_), and NanoJagg/ICG spectra (Figure S33b), revealed that in non‐senescent tumors, NanoJaggs colocalise with Hb and HbO_2_ indicating their distribution within vasculature (Figure 5d). However, in case of Palbociclib‐treated (senescent) xenografts, NanoJaggs were found to infiltrate the tumor tissue, and there was no overlap with Hb and HbO_2_ signals indicating that they were not co‐ localized with vasculature. To validate that the increase in NanoJagg signal is not due to increased blood perfusion within the tumor, a comparison of HbO_2_ and Hb signals was conducted between senescence and non‐senescent tumors (Figure S33c). This analysis revealed no significant differences between two groups (p=0.5099 and p=0.2926 respectively).

To further confirm that the NanoJagg signal is associated with senescence, a correlation analysis was conducted using parameters such as the tumor size and growth rate (Figure S33d). Remarkably, we found that the photoacoustic NanoJagg signal strongly correlates with the arrested tumor growth rate (r^2^=0.875, p=0.0061), but not the tumor size (r^2^=0.015, p=0.817, ns). This correlation with growth rate was not observed in the case of endogenous hemoglobin HbO_2_ (r^2^=0.112, p=0.516, ns, Figure S33e).

The presence of NanoJaggs in senescent tumors was additionally confirmed by fluorescence imaging of the ex vivo tumor slices (Figure 5e). The total number of NanoJagg‐positive cells is significantly increased in senescent tumors compared to control counterparts (p=0.0002). Moreover, these positive cells within the senescent tumors displayed a significantly increased NanoJagg fluorescence signal when compared to the untreated controls (p=0.0301, Figure S33f).

Finally, PA imaging was also employed to monitor NanoJaggs biodistribution and routes of elimination confirming the data obtained from ex vivo studies (Figure S34a). Strong PAT signal in kidneys suggests renal clearance that is in line with the fluorescence ex vivo and in vivo data. Contrary to the ex vivo images, NanoJagg accumulation can also be observed in the spleen and liver, possibly due to the improved sensitivity of the imagng technique. However, importantly, NanoJaggs signal in the liver, spleen and kidneys decreases over time, and becomes significantly reduced after 24 h (Figure S34a and b). Furthermore, no discernible histological differences in tissue architecture between mice treated with the vehicle and those treated with NanoJaggs were revealed by hematoxylin and eosin (H&E) staining, indicating an absence of hepatic and renal damage, as well as the lack of inflammation in spleen (Figure S34c).

## Conclusions

The organic nanostructured J‐aggregates (NanoJaggs) described in this study enable the detection of various types of chemotherapy‐induced senescence using range of techniques such as flow cytometry, confocal imaging, in vivo fluorescence, and PA imaging across different settings, including in vitro, ex vivo, and in vivo models. NanoJagg synthesis is highly reproducible and scalable and delivers a probe, which is stable in water and biologically relevant media. The probe is also suitable for lyophilization, which significantly improves storage time and transport. Unlike previously reported J‐aggregate nanostructures, our probe has high purity and we have shown that it is composed completely of ICG dimers. Although further research is needed to thoroughly understand the mechanism of NanoJagg uptake and lysosomal targeting, we provide evidence that the preferential uptake of NanoJaggs in senescence is regulated, at least in part, by clathrin‐mediated endocytosis and macropinocytosis. We also demonstrate that the enhanced accumulation of the probe in senescent cells correlates with their increased lysosomal content.

The simple synthesis, specificity towards senescent cells and strong PAT signal, makes NanoJaggs suitable for large‐scale preparation and clinical translation. However, further studies are needed, including a thorough biodistribution and biodegradation study in healthy mice as well as the investigation of other models such as senescence‐related chronic disorders, oncogene‐induced senescence, and naturally aged mice.

Our study has demonstrated that NanoJaggs can effectively serve as selective contrast agents for fluorescence and PA imaging of senescent cells. The properties of NanoJaggs coupled with enhanced tissue penetrance of PA imaging, offer a practical avenue for translation of this senoprobe into human settings. The in vivo detection and monitoring of the senescent burden using NanoJaggs could not only offer valuable insights into the extent of tissue dysfunction but could also greatly impact risk stratification and diagnostic efficiency for patients across various diseases including cancer. Assessment of senescent burden could aid differentiation of patients responding well to the therapy and those at risk of tumor relapse and metastasis. In addition, in vivo detection tool could provide data on the efficiency of developed senolytic interventions aimed at targeted removal of senescent cells. Indeed, the demonstrated increase in NanoJaggs uptake by senescent cells could lead to the design of theranostic tools or selective nanostructured senolytics that do not require additional functionalization with targeting species.

## Supporting Information

The authors have cited additional references within the Supporting Information (Ref. [30,31]).

## Conflict of interests

AGB, DME and LF have filed a patent for the presented technology.

1

## Supporting information

As a service to our authors and readers, this journal provides supporting information supplied by the authors. Such materials are peer reviewed and may be re‐organized for online delivery, but are not copy‐edited or typeset. Technical support issues arising from supporting information (other than missing files) should be addressed to the authors.

Supporting Information

## Data Availability

The data that support the findings of this study are available in the supplementary material of this article.
