# Supplementary material for: An Indocyanine Green‐Based Nanoprobe for In Vivo Detection of Cellular Senescence
Source: Angew Chem Int Ed Engl. 2024 May 16;63(25):e202404885. doi: 10.1002/anie.202404885 (PMC11497227; doi:10.1002/anie.202404885)
Supplement: Supplementary file 1 — Supporting Information [file ANIE-63-e202404885-s001.pdf]

Angewandte Chemie
Eine Zeitschrift der Gesellschaft Deutscher Chemiker

Supporting Information

An Indocyanine Green-Based Nanoprobe for *In Vivo* Detection of Cellular Senescence

A. G. Baker, M. Hartono, H.-L. Ou, A. B. Popov, E. L. Brown, J. Joseph, M. Golinska, E. González-Gualda, D. Macias, J. Ge, M. Denholm, S. Morsli, C. Sanghera, T. R. Else, H. F. Greer, A. Vernet, S. E. Bohndiek, D. Muñoz-Espín, L. Fruk**

Supporting Information
©Wiley-VCH 2021
69451 Weinheim, Germany

Indocyanine Green-based Nanoprobe for *In Vivo* Detection of Cellular Senescence

Andrew G. Baker, Muhamad Hartono, Hui-Ling Ou, Andrea Bistrović Popov, Emma L. Brown, James Joseph, Monika Golinska, Estela González-Gualda, David Macias, Jianfeng Ge, Mary Denholm, Samir Morsli, Chandan Sanghera, Thomas R. Else, Heather F. Greer, Aude Vernet, Sarah E. Bohndiek, Daniel Muñoz-Espín*, Ljiljana Fruk*

Abstract: There is an urgent need to improve conventional cancer-treatments by preventing detrimental side effects, cancer recurrence and metastases. Recent studies have shown that presence of senescent cells in tissues treated with chemo- or radiotherapy can be used to predict the effectiveness of cancer treatment. However, although the accumulation of senescent cells is one of the hallmarks of cancer, surprisingly little progress has been made in development of strategies for their detection *in vivo*. To address a lack of detection tools, we developed a biocompatible, injectable organic nanoprobe (NanoJagg), which is selectively taken up by senescent cells and accumulates in the lysosomes. The NanoJagg probe is obtained by self-assembly of indocyanine green (ICG) dimers using a scalable manufacturing process and characterized by a unique spectral signature suitable for both photoacoustic tomography (PAT) and fluorescence imaging. *In vitro*, *ex vivo* and *in vivo* studies all indicate that NanoJaggs are a clinically translatable probe for detection of senescence and their PAT signal makes them suitable for longitudinal monitoring of the senescence burden in solid tumors after chemotherapy or radiotherapy.

Table of Contents

Content	Page
**1. Materials and Methods**	2
1.1. Synthesis of NanoJagg probe	2
1.2. NanoJagg characterization	3
1.3. Cryo-TEM of NanoJagg probes	3
1.4. Photoacoustic properties of NanoJaggs	3
**2. *In vitro* materials and methods**	3
2.1. Generation of control and senescent cell lines	3
2.2. Immunoblotting	3
2.3. Senescence Associated β-Galactosidase (SA-β-gal) staining	3
2.4. Flow cytometry	4
2.5. Confocal microscopy	4
2.6. Organelle colocalization analysis by confocal microscopy	4
2.7. Endocytosis analysis by confocal microscopy	4
**3. *In vivo* material and methods**	5
3.1. Mice studies	5
3.2. *Ex vivo* imaging by IVIS	5
3.3. *In vivo* photoacoustic imaging	5
3.4. Immunohistochemistry and immunofluorescence	5
**4. Statistical Analysis**	6
**5. Results and Discussion**	7
5.1. NanoJagg chemical characterization	7
5.2. NanoJagg *in vitro* evaluation	14
5.3. NanoJagg endocytosis studies	27
5.4. NanoJagg *ex vivo evaluation*	33
5.5. NanoJagg *in vivo* evaluation with fluorescent imaging	34
5.6. NanoJagg *in vivo* evaluation with photoacoustic tomography	35
**6. Author Contributions**	38

1.Material and Methods

1.1. Synthesis of NanoJagg probes

Aqueous ICG (Acros Organics, 10321541) solution (0.75 mM, 10 mL) was sonicated for 10 min and then heated to 65ºC under stirring (500 rpm). Formation of J-aggregates (λ_{max}=895 nm) was monitored by UV-Vis spectrophotometer indicating the completion of reaction at 24 h, after which the reaction mixture was centrifuged and washed three times (17000 rpm/ 31000 g at 4ºC for 30 min in a Sorvall LYNX 4000 high speed centrifuge). The pellet was redispersed in deionized water, filtered through a 0.2 μm filter, and lyophilized to obtain dark green NanoJagg NPs (5.4 mg, 54% yield). Lyophilization was carried out using a Telstar LyoQuest benchtop freeze dryer (0.008 mBar, −70 °C).

1.2. NanoJagg characterization

UV-Vis absorption spectra were obtained with an Agilent Cary 300 Spectrophotometer, as well as, The Spark (TECAN), and Infinite 200 Pro (Tecan). Fluorescence emission spectra were obtained using a Varian Cary Eclipse Fluorescence Spectrophotometer as well as, The Spark (TECAN), and Infinite 200 Pro (Tecan). The hydrodynamic size and zeta potential of the NanoJagg NPs were measured using a Zetasizer Nano Range instrument (Malvern Panalytical).

To estimate the extinction coefficient for the concentration of NanoJagg NPs, we assumed spherical particles, partial overlap of the ICG dimer within the structure, and uniform internal molecule structure. We used the dimer of ICG's molecular weight of 1502.56 Da and size was measured in PyMOL (Schrödinger) to first determine the number of dimers in the solution and then size. We then adjusted this concentration, based on the number of dimers per NanoJagg NP which we estimated to be ~5,700 per nanoparticles.

To determine the chemical composition LC-MS and ^{1}H NMR were conducted on the obtained NanoJaggs and the supernatant obtained during the centrifugation. LC-MS was performed using a Waters' Xevo G2-S bench top QTOF and was performed by the Department of Chemistry Mass Spectrometry Service, University of Cambridge (UK). Samples were first dissolved in methanol to ensure the NanoJagg structure was disassembled. ^{1}HNMR measurements were carried out using 400 MHz QNP Cryoprobe Spectrometer (Bruker)

by the NMR service of the Department of Chemistry, University of Cambridge. Samples were dissolved in deuterated methanol to make sure the NanoJagg structure was dissolved.

ICG: ^{1}H NMR (400 MHz, MeOD): δ 8.23 (d, J = 8.6 Hz, 1H), 8.13 – 7.94 (m, 2H), 7.70 – 7.56 (m, 1H), 7.54 – 7.41 (m, 1H), 6.73 – 6.49 (m, 1H), 6.39 (d, J = 13.4 Hz, 1H), 4.25 (t, J = 6.5 Hz, 1H), 2.94 (t, J = 6.8 Hz, 1H), 2.25 – 1.74 (m, 6H). HRMS: calculated for C43H47N2O6S2- $(M+H)^+$: Mass predicted:752.29; Found:752.2930

NanoJagg (pellet): ^{1}H NMR (400 MHz, MeOD) δ 8.37 – 8.25 (m, J = 15.7, 8.0 Hz, 4H), 8.10 – 7.92 (m, 6H), 7.67 (dd, J = 18.5, 8.4 Hz, 4H), 7.56 – 7.43 (m, 4H), 6.53 – 6.41 (m, 2H), 5.91 (d, J = 13.9 Hz, 1H), 4.30 – 4.21 (m, 2H), 4.14 – 4.06 (m, J = 7.4 Hz, 2H), 4.02 – 3.92 (m, 2H), 2.91 – 2.75 (m, J = 14.8, 8.2 Hz, 5H), 2.14 (s, 5H), 2.07 (d, J = 8.2 Hz, 6H), 2.00 – 1.79 (m, 9H). HRMS: calculated for C86H92N4O12S42- $(M+H)^+$: Mass predicted:751.28; Found:751.2880

Supernatant obtained from the reaction mixture after purification: ^{1}H NMR (400 MHz, MeOD) δ 8.38 – 8.20 (m, 1H), 8.19 – 8.11 (m, 1H), 8.08 – 7.87 (m, 2H), 7.84 – 7.76 (m, 1H), 7.74 – 7.57 (m, 1H), 7.56 – 7.39 (m, 1H), 7.40 – 7.32 (m, 1H), 6.52 – 6.33 (m, 1H), 3.94 – 3.85 (m, 1H), 3.03 – 2.70 (m, J = 31.1, 24.2, 17.8, 10.9 Hz, 2H), 2.31 – 1.71 (m, 6H), 1.68 – 1.55 (m, J = 7.7 Hz, 1H), 1.38 – 1.08 (m, 1H).

1.3. Cryo-TEM of NanoJagg probes

Cryo-TEM micrographs were obtained using a Thermo Scientific (FEI Company) Talos F200X G2 microscope operated at 200 kV. Images were recorded on a Ceta 4k x 4k CMOS camera and processed with Velox software. Specimens for investigation were prepared through vitrification by plunge freezing of the aqueous suspensions on copper grids (300 mesh) with lacey carbon film (Agar Scientific). Prior to use, the grids were glow discharged using a Quorum Technologies GloQube instrument at a current of 25 mA for 60 s. Suspensions of the samples (2.5 µL of a 1 mg/mL solution) were pipetted onto the grid, blotted using filter paper, and immediately frozen by plunging in liquid ethane utilizing a fully automated and environmentally controlled blotting device, Vitrobot Mark IV. The Vitrobot chamber was set to 4˚C and 95% humidity. Samples after vitrification were kept under liquid nitrogen until they were inserted into a Gatan Elsa cryo holder and analyzed in the TEM at −178 °C. Cryo-TEM was performed by Heather Greer of the Department of Chemistry Electron Microscopy Facility.

1.4. Photoacoustic properties of NanoJaggs

Photoacoustic measurements were performed using a commercial PAT system (inVision256-TF; iThera Medical GmbH) and reconstructed using a linear model-based approach in their proprietary software. A tube of NanoJaggs was placed in the center of a tissue-mimicking phantom that mimics the optical and acoustic properties of biological tissues.[64] A region of interest was drawn around the NanoJagg inclusion, and the mean photoacoustic signal was extracted at each wavelength. After matching I_{max} UV-Vis for both to 1AU, the photoacoustic signals of the ICG monomer and J-aggregate were compared, using the following wavelengths: 700, 720, 740, 760, 770, 780, 790, 800, 820, 840, 860, 870, 880, 890, 895, 900, 905, 910, 920, and 940 nm.

2. *In vitro* materials and Methods

2.1. Generation of control and senescent cell lines

The A549 (human lung adenocarcinoma) cell line was obtained from the European Collection of Authenticated Cell Cultures (ECACC). The SK-MEL-103 (human melanoma) cancer cell line was acquired from the American Type Culture Collection (ATCC). These cell lines were maintained in DMEM (Sigma) and supplemented with 10% FBS. For senescence induction, SK-MEL-103 cells were supplemented with the same media containing Palbociclib (PD0332991, MCE.) at 5 µM for 7 days. A549 cells were supplemented with the same media containing 15µM cisplatin (Stratech) for 10 days or 10 µM Palbociclib (MCE, PD0332991) for 10 days. Wi-38 cells were purchased from American Type Culture Collection (ATCC). This cell line was maintained in MEM (Thermo Fisher) supplemented with 10% FBS, 2 mM l-Glutamine and 1 mM sodium pyruvate (Sigma). Wi-38 cells were induced with 10Gy of X-ray irradiation, then maintained for 10 days. Human umbilical vein endothelial cells (HUVECs), a primary endothelial cell line, were cultured in F-12K Medium (Gibco) supplemented with 10% FBS, 0.1 mg/mL heparin (Sigma) and 0.03 mg/mL endothelial cell growth supplement from bovine neural tissue (ECGS; Sigma). Mouse embryonic fibroblasts were maintained in DMEM supplemented with 10% FBS. All cell lines were incubated in 20% O_2 and 5% CO_2 at 37 °C. Cells were routinely tested for mycoplasma using the universal Mycoplasma Detection Kit (ATCC) or by RNA-capture ELISA. For experiments with cells, Cisplatin (Selleck Chemicals, S1166) was reconstituted in sterile PBS; Palbociclib (MCE, PD0332991) was reconstituted in DMSO.

2.2. Immunoblotting

Cell lysis was performed using RIPA buffer (Sigma) supplemented with phosphatase inhibitors (PhosSTOP™ EASYpak Phosphatase Inhibitors Cocktail, Roche) and protease inhibitors (cOmplete™ Protease Inhibitor Cocktail, Roche). Proteins were quantified and

separated by SDS-PAGE and transferred to polyvinylidene difluoride (PVDF) membranes (Millipore) according to standard protocols. Membranes were immunoblotted with antibodies against p21 (556430) from BD Pharmingen, phospho-Rb (pRBS807/822) from Cell Signalling and normalized to GAPDH (ab9485) from Abcam. After incubation with the primary antibody overnight, membranes were washed and incubated with secondary HRP-conjugated AffiniPure antibodies (Jackson ImmunoResearch) for 1 h at room temperature and subsequently incubated with Enhanced Chemiluminescence Detection solution (Amersham).

2.3. Senescence Associated β-Galactosidase (SA-β-gal) staining

SA-β-gal staining was performed using the Senescence β-galactosidase Staining kit (Cell Signalling), following the manufacturer instructions. Briefly, cells were fixed at RT for 15 min with a 2% formaldehyde, washed with PBS and incubated overnight at 37 °C with the staining solution containing X-gal in N,N-dimethylformamide (pH 6.0 adjusted with HCl). The next day cells were washed 3x with PBS for 2 min, and finally PBS was placed over the cells for imaging. Pictures were taken using a Wide Field Zeiss Axio Observer 7. For tissue cryosections this method was repeated, however the tissue was incubated with x-gal for 4–6 h.

2.4. Flow cytometry

Flow cytometry was measured on an LSR Fortessa (BD - Becton Dickinson). For the uptake of NanoJaggs we used 6 well plates and for all cell lines used we seeded 200,000 cells/well. Once the cells were attached, culture medium was changed to DMEM supplemented with 0.2 % FBS and cells were incubated with the NanoJaggs for 12–16 h. The cells were trypsinized and resuspended in PBS buffer with 2% FBS. DAPI (Sigma Aldrich, D9542) 0.1 µg/mL was added to each sample to exclude dead cells. For each condition 10,000 live events were collected for each. The analysis of all flow cytometry data was performed using FlowJo v10 (Treestar, OR). Unstained control and senescent cells were used as reference for each independent experiment. For ICG the 640 nm laser was used with an emission window of 750–810 nm. For the NanoJaggs, the 640 nm laser was used with an emission window of 708–753 nm.

2.5. Confocal microscopy

Cells were seeded in a 96-well plates m-clear plates (Greiner Bio-One #655087) 3,500 control cells/well and 5,000 senescent cells/well. After 24 h cells were incubated with NanoJaggs at concentrations of 10-100 µg/mL. Confocal images were acquired on a Leica SP5 confocal microscope using a 20X HCX PL APO 0.5 NA dry objective or a 40X HCX PL APO 1.3 NA oil immersion objective. Hoechst (ThermoFisher, H1399) was used to specifically dye the nucleus at 5 µg/mL. Lysotracker (Cell Signalling Technology, FM8783), Mitotracker (MitoTracker Green (Cell Signalling Technology, FM9074), and ERtracker (BODIPY™ FL Glibenclamide, Thermo Fisher, E34251) were detected by using excitation wavelength of 488 nm (Argon laser) and with a detection window between 510 and 530 nm. The organelle specific dyes were used according to their manuals. NanoJaggs were detected by using excitation wavelength of 633 nm (Argon laser) and with a detection window between 680–720 nm. Cells without dyes and NanoJaggs were images as autofluorescence controls using the corresponding excitation and detection wavelengths. Images were analyzed with LAS AF Lite (Leica).

2.6. Organelle colocalization analysis by confocal microscopy

Cells were trypsinized and seeded in a flat-bottom µ-clear 96-well plates (Greiner Bio-One, #655087) at a density of 3,500–5,000 control and 4,000–6,000 senescent cell/well. Once the cells were attached, culture medium was changed to DMEM supplemented with 0.2% FBS and incubated with NanoJaggs (100 µg/mL – 10 µg/mL) for 12–16 h. Afterward, cells were washed 3x with PBS for 5 min. Specific organelle stains LysoTracker green DND-26 (Cell Signalling Technology, FM8783), MitoTracker Green (Cell Signalling Technology, FM9074), ER-Tracker™ Green (BODIPY™ FL Glibenclamide, Thermo Fisher, E34251) were used according to the manufacturers protocol. For nuclei staining, Hoechst (ThermoFisher, H1399) at 5 µg/mL in PBS was added 10 min prior to analysis. Colocalization analysis was performed using ImageJ (Fiji) Coloc2.

2.7. Endocytosis analysis by confocal microscopy

Cells were trypsinized and replated in flat-bottom µ-clear 96-well plates (Greiner Bio-One, #655087). Cells were seeded at a density of 3,500-5,000 control and 4,000-6,000 senescent cells per well. Once cells were attached, culture medium was changed to DMEM without FBS and exposed to several different inhibitors for 1 h. Pitstop2 (10 µM, Abcam ab120687), Dyngo4a (10 µM abcam. ab120689), Chloroquine (50 µM, Tocris, 4109), Prochlorperazine (15 µM, Sigma, P9178) and LY2940020 (20–50 µM, Sigma, 440202). After incubation, NanoJaggs were added in DMEM supplemented with 0.2% FBS, in a range from of 10 to 100 µg/mL for 16 h. Afterwards cells were washed 3x with PBS for 5 min. Specific organelle stains lyso green DND-26 (Cell Signalling Technology, FM8783) was used according to their manuals. For nuclei staining, Hoescht (ThermoFisher, H1399) at 5.0 ug/mL in PBS was added 10 min prior to analysis.

3. *In vivo* materials and Methods

3.1. Mice Studies

All mice were treated in strict accordance with the local ethical committee (University of Cambridge License Review Committee) and the UK Home Office guidelines. Experiments involving mice were performed under project licences P7EC604EE and PP7061972 held by D.M-E., and PE12C2B96 held by S.E.B, all issued by the Home Office, UK. Studies were reviewed and approved for ethical conduct by the Central Biomedical Services (CBS) and the CRUK Cambridge Institute local animal welfare and ethical review bodies, regulated under the Animals (Scientific Procedures) Act 1986 (ASPA). All studies were conducted in compliance with The International Guiding Principles for Biomedical Research involving Animals. Tumor xenografts were established using SK-MEL-103, a melanoma cell line, and A549 a lung carcinoma cell line. Cells were trypsinized, counted with a hemocytometer, and injected subcutaneously ($0.5x10^6$ cells for SK-MEL-103 cells in a volume of 100 µL) per dorsolateral flank in 8– to 10–week-old athymic nude female mice (Hsd:Athymic Nude-Foxn1nu) purchased from Charles River, for A549 cells a cell suspension was prepared by mixing Matrigel (Corning) 1:1 with $3x10^6$ A549 cells and injected per dorsolateral flank in 8– to 10–week-old athymic nude female mice (Hsd:Athymic Nude-Foxn1nu) purchased from Charles River . Tumor volume was measured every 2 days with a caliper and calculated as $V = (a \times b2)/2$ where a is the longer and b is the shorter of two perpendicular diameters. Palbociclib (MCE, PD0332991). was dissolved in 50 mM sodium lactate at 10.0 mg/mL and administered by daily oral gavage at the indicated doses. Cisplatin (Stratech) was dissolved in saline (1mg/kg) and delivered three times per week by intraperitoneal injection. 200 µL of NanoJaggs (1 mg/mL) and 200 µL of ICG (1 mg/mL) were injected via the tail vein in clear DMEM (ThermFisher, no phenol red).

3.2 Ex vivo and *in vivo* imaging by IVIS

An IVIS Spectrum Imaging System (Perkin Elmer Inc) was used for *ex vivo* and *in vivo* fluorescence imaging. For *in vivo* imaging mice were imaged both before and after NanoJagg injection via the tail vein. For *ex vivo* imaging, mice were sacrificed by cervical dislocation and organs and tumor xenografts were analyzed immediately after harvesting. ICG was detected using an excitation wavelength of 710–740 nm and emission bandpass from 810–830. NanoJaggs were detected using an excitation wavelength of 605 nm (bandpass ±30nm) and emission of bandpass of 700–720 nm. Fluorescence imaging quantification was performed by Living Image 3.2 software (Perkin Elmer Inc). A region of interest area (ROI) was drawn over around the tumors. Fluorescence activity is measured as average Radiant Efficiency with the equation of of $[p/s/cm2/sr]/[\mu W/cm2]$.

3.3. In vivo Photoacoustic Imaging

Photoacoustic measurements were performed using a commercial PAT system (inVision256-TF; iThera Medical GmbH). Briefly, a tunable (660–1,300 nm) optical parametric oscillator, pumped by a nanosecond (ns) pulsed Nd:YAG laser, with 10 Hz repetition rate and up to 7 ns pulse duration is used for signal excitation. All mice were treated in strict accordance with the local ethical committee (University of Cambridge License Review Committee) and the UK Home Office guidelines. Tumor xenografts were established using SK-MEL-103 melanoma cells. Cells were trypsinized, counted with a haemocytometer, and injected subcutaneously (0.5*10^6 cells in a volume of 100 µL per dorsolateral flank) in 8– to 10–week–old athymic nude female mice (Hsd:Athymic Nude-Foxn1nu, Charles River). Tumor volume was measured every 2 days with a caliper and calculated as $V = (a \times b2)/2$, where a is the longer and b is the shorter of two perpendicular diameters. Palbociclib (MCE, PD0332991) was dissolved in 50 mM sodium lactate at 10 mg/mL and administered by daily oral gavage at the indicated doses. 200 µL of NanoJaggs (1 mg/mL) and 200 µL of ICG (1 mg/mL) were injected by the tail vein in DMEM (Thermo Fisher, no phenol red).

Mice were anaesthetized using <3% isoflurane and placed in a custom animal holder (iThera Medical), wrapped in a thin polyethylene membrane, with ultrasound gel (Aquasonic Clear, Parker Labs). Mice were imaged using the wavelengths 700, 720, 740, 760, 780, 790, 800, 820, 840, 860, 870, 880, 890, 895, 900, 905, 910, 920, and 940 nm, with an average of 10 pulses per wavelength.

PAT data analysis was performed using ViewMSOT software (v3.6.0.119; iThera Medical GmbH). Model-based image reconstruction and linear spectral unmixing were applied on data in the 700–940 nm wavelength range to retrieve the relative signal contributions of oxy–(HbO2), deoxy–(Hb) hemoglobin, ICG and the NanoJaggs. Linear spectral unmixing was performed with published spectra for ICG, oxyhemoglobin, and deoxyhemoglobin as well as the NanoJaggs absorption spectrum collected in this study. Regions of interest were drawn manually over the tumor area, and unmixing quantities were averaged over the entire tumor volume. A corresponding background region of interest was drawn near the back of the mouse for each anatomical plane.

Signal to background ratio was taken as the signal from the tumor ROI divided by the signal of the background ROI for each anatomical slice: $SBR = \sum \frac{Tumor\ ROI}{Background\ ROI}$. Contrast to noise ratio was calculated as the sum of the differences of the ROI signal and background divided by the standard deviation of the background signal for each tumor slice: CNR=$\sum \frac{Tumor\ ROI - Background\ ROI}{\sigma\ Background\ ROIs}$.

3.4 Immunohistochemistry and Immunofluorescence

For Immunohistochemistry, tumors and organs were extracted and put in 10% neutral buffered formalin (4% formaldehyde in solution). They were then transferred to 70% ethanol. The samples were then embedded in paraffin by the Histopathology Core Facility of CRUK Cambridge Institute, and sent for processing at CNIO, where they were stained using Phospho-Rb (Ser807/811), and Ki-67 (D3B5). Digital image analysis was performed using HALO and the CytoNuclear v2.0.9 imaging module (Indica Labs, Albuquerque, USA).

Immunofluorescence was performed on samples previously placed in OCT and frozen. 10 μm slices were made using a Leica CM3050 S cryostat. Briefly, samples were washed twice with PBS, fixed in 4% PFA solution for 10 min, washed again with PBS, and then permeabilized with 0.25% Triton X-100. Samples were then blocked for 1 h using 2% normal donkey serum. In the same blocking solution, a 1/500 dilution of the primary antibody for Ki-67 Rabbit host (ICH-00375) from Bethyl Laboratories. After incubation overnight at 4 °C, slides were washed twice in PBS and anti-rabbit secondary was incubated for 2 h at RT. Alexa Fluor® 488 AffiniPure Donkey Anti-Rabbit IgG was used (711-545-152) from Jackson Immuno Research. Slides were then mounted using fluoromount-G (0100-01) from SouthernBiotech and imaged using the Leica SP5 confocal microscope using a 20× HCX PL APO 0.5 NA dry objective or a 40× HCX PL APO 1.3 NA oil immersion objective. Images were analyzed with LAS AF Lite (Leica).

4. Statistical Analysis

All analysis was performed unblinded. Statistical analyses were performed as described in the figure legend for each experiment. Statistical significance was determined by Student's t tests (two-tailed) using Prism 9 software (GraphPad) as indicated. A p-value below .05 was considered significant and indicated with asterisk: *$p < 0.05$, **$p < 0.01$, ***$p < 0.001$, and ****$p < 0.00001$.

5. Results and Discussion

5.1. NanoJaggs: chemical characterization

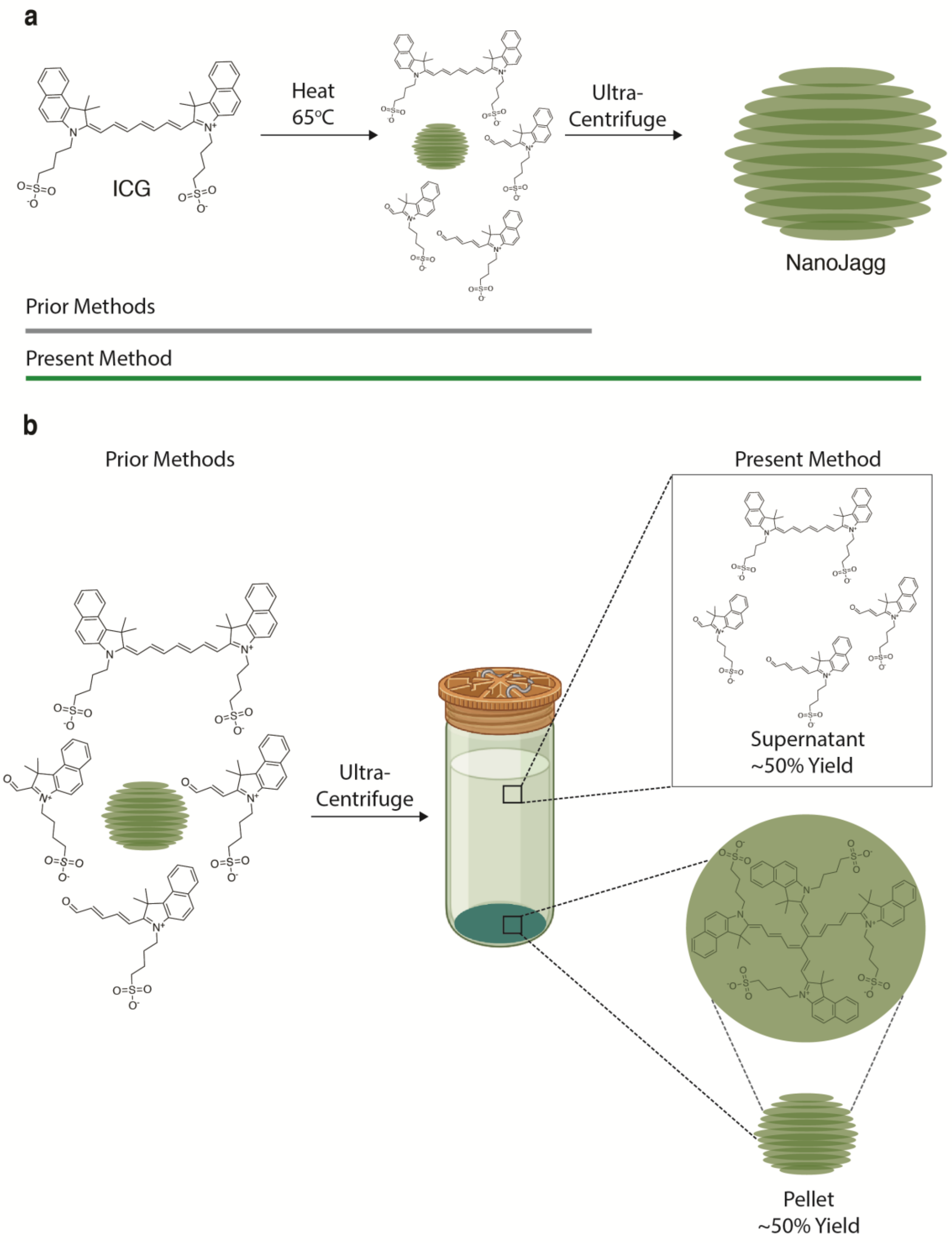

Figure S1. Preparation of NanoJaggs and some of the side products obtained in previously reported methods. (a) Prior reported methods do not report purification step. In addition, solutions were not fully characterized. Introduction of centrifugation step results in pure NanoJaggs. (b) Some of the side products identified in the reaction mixture. After centrifugation, a dark green pellet obtained represents ~50% by mass yield of the reaction and contains NanoJaggs, J-aggregate nanoparticles, composed of a dimer of ICG as opposed to what was previously believed to be a monomer.

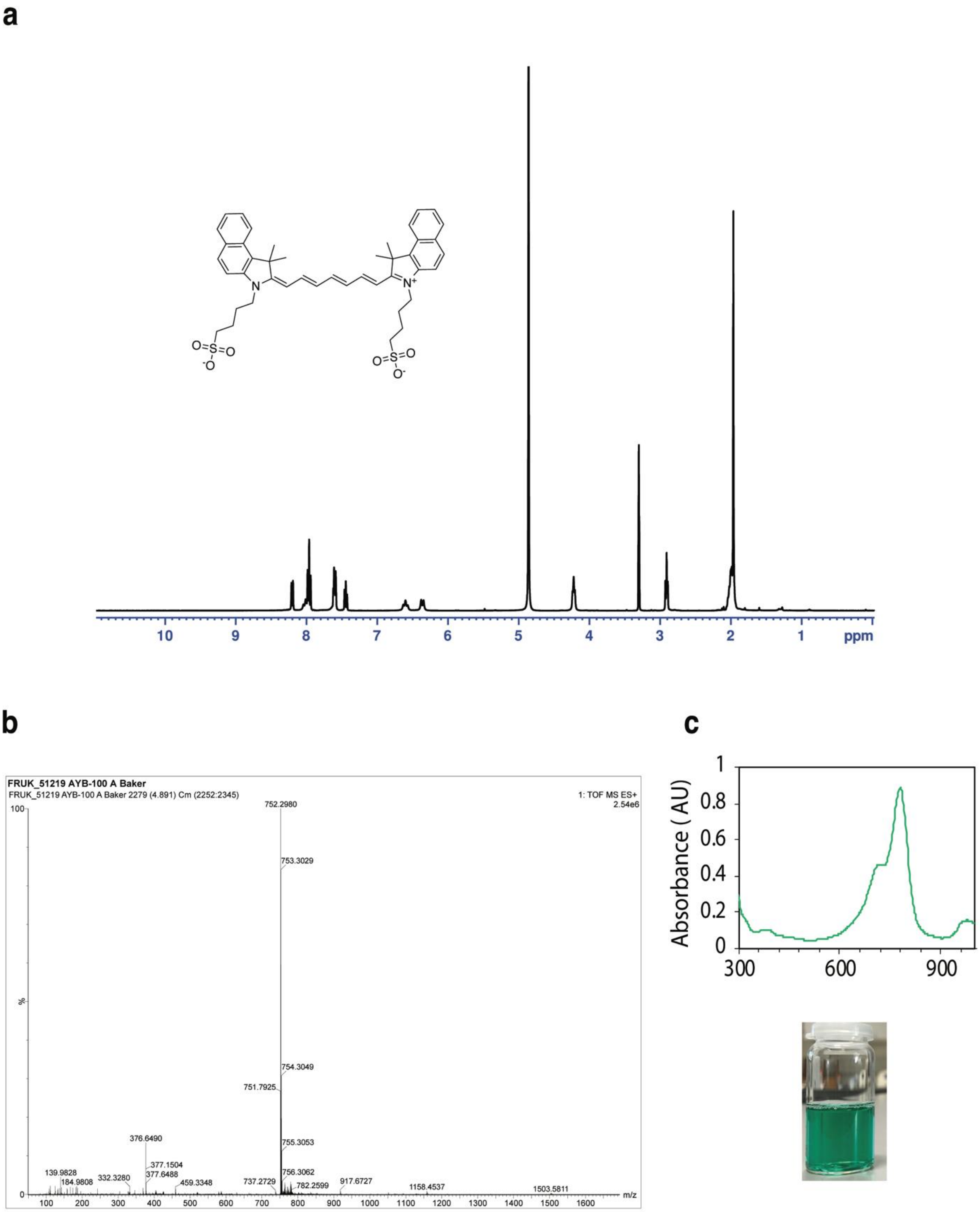

Figure S2. Indocyanine Green Dye (ICG) characterization. (a) ^{1}H NMR spectrum, (b) LC-MS/MS, and (c) UV-Vis of green ICG solution. To obtain the NMR spectrum, 10 mg of ICG is dissolved in deuterated methanol (CD3OD), while mass spectrometry was performed using ICG in methanol.

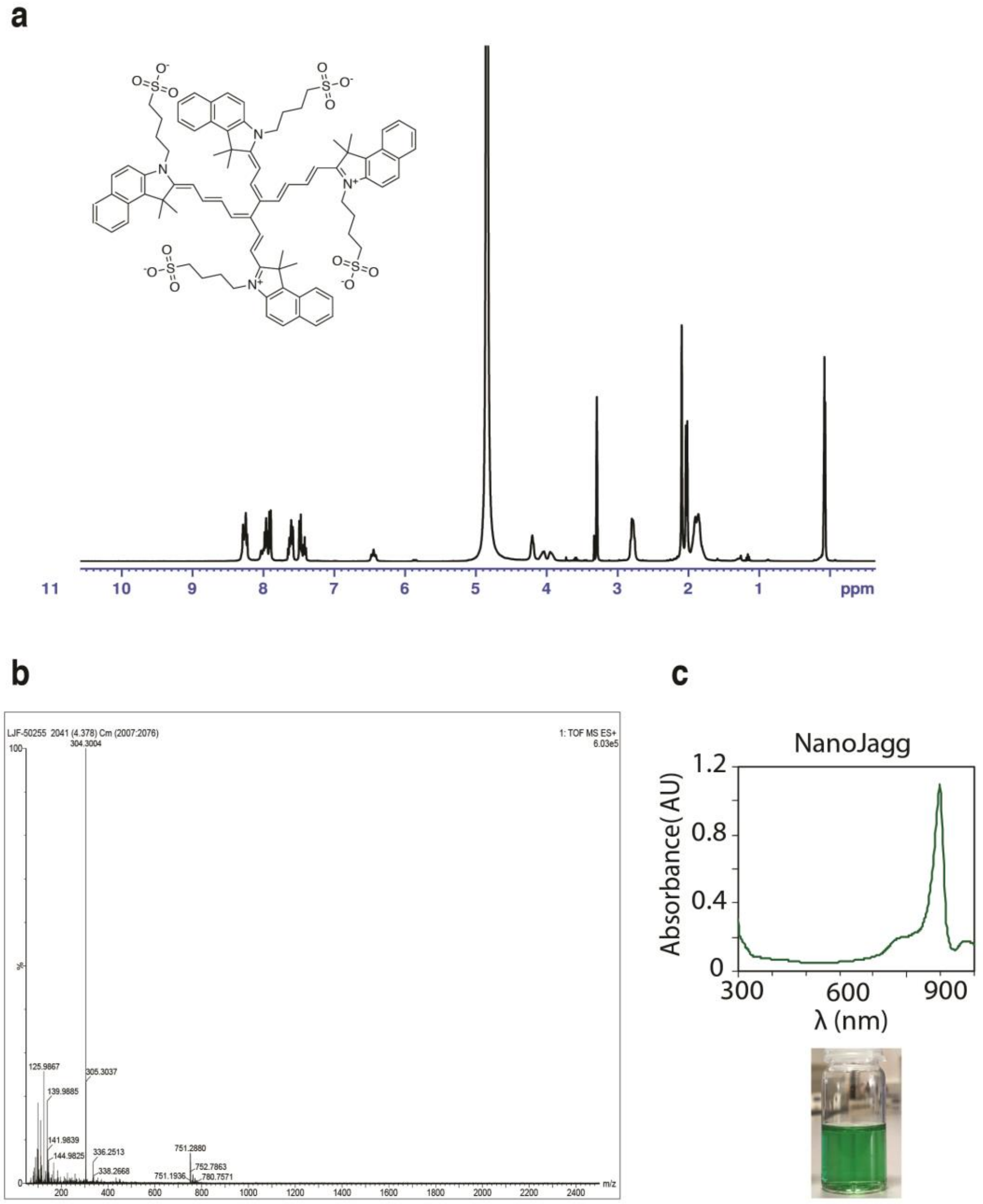

Figure S3. Characterization of NanoJagg pellet. (a) ^{1}H NMR spectrum, (b) LC-MS/MS data, and (c) UV-VIS spectrum. ^{1}H NMR was obtained using 10 mg of the pellet dissolved in deuterated methanol (CD3OD) while pellet dissolved in methanol was used for mass spectrometry.

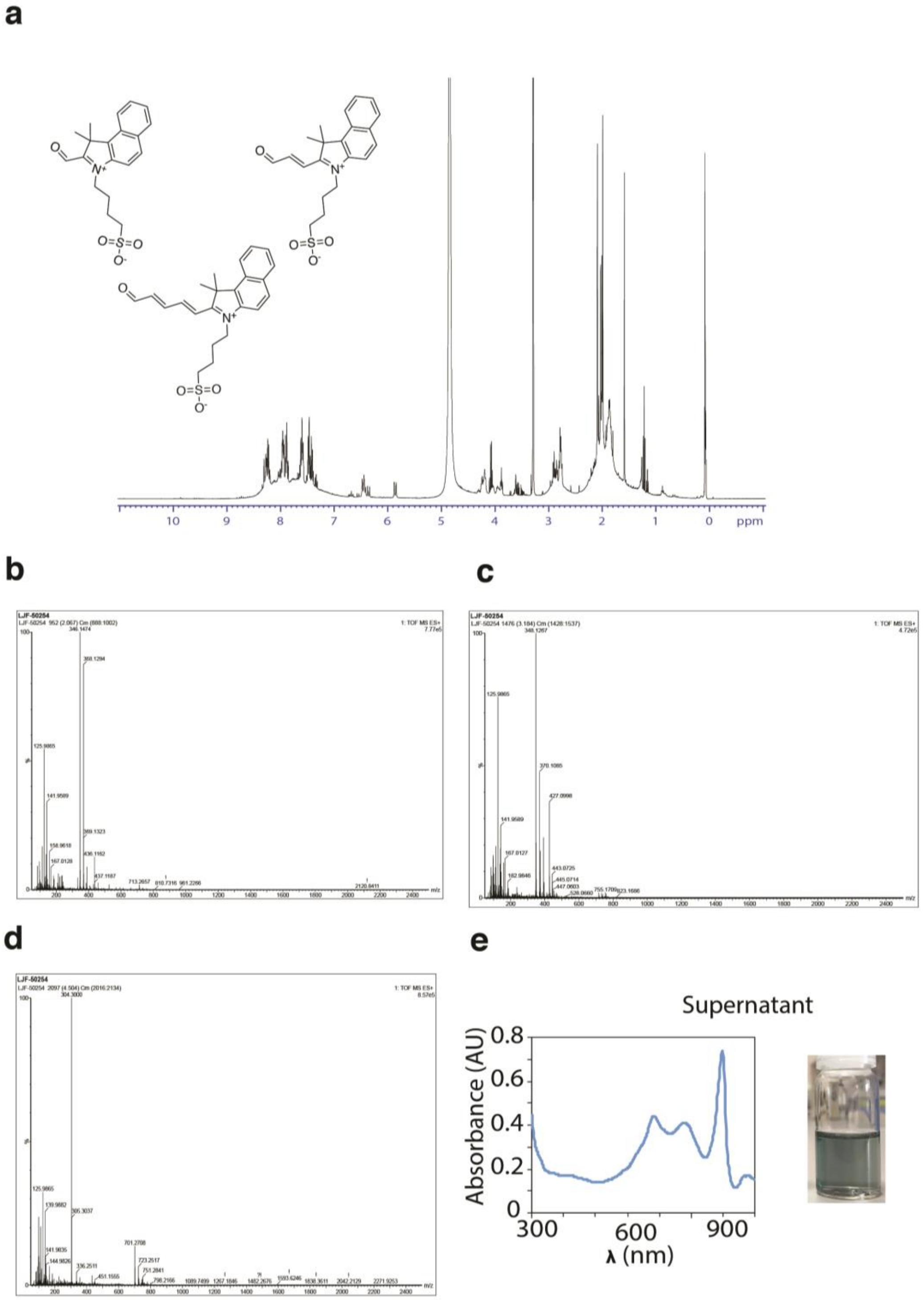

Figure S4. Characterization of the supernatant. (a) ^{1}H NMR spectrum, (b, c, d) LC-MS/MS data, and (e) UV-VIS spectrum including the photo of the supernatant. ^{1}H NMR was obtained using deuterated methanol (CD3OD).

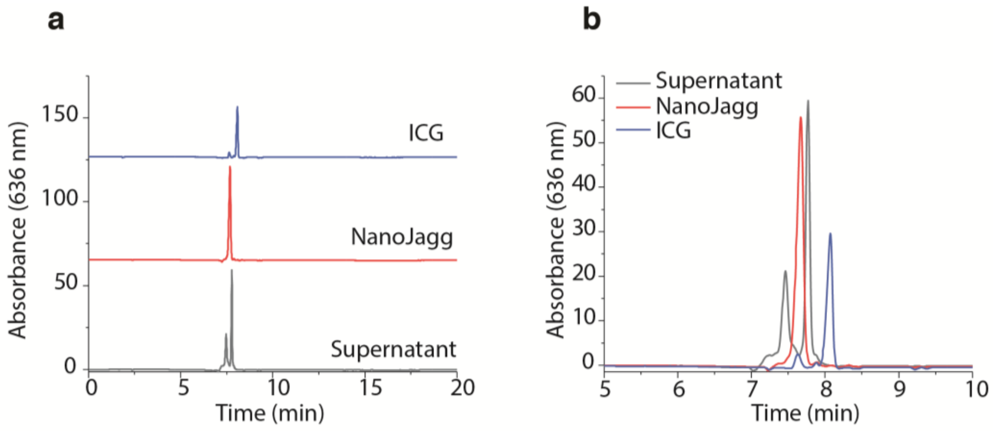

c

Compound	Retention Time (min)	Area (%)
ICG	7.63	8.5
	7.89	3.1
	8.07	88.4
NanoJagg	7.67	97.2
	7.89	2.8
Supernatant	7.45	44.3
	7.76	55.7

Figure S5. HPLC traces and retention times of ICG, NanoJaggs and supernatant. Individual (a) and combined (b) HPLC traces of ICG, NanoJaggs and supernatant illustrating the differences in retention times. (c) Retention times of individual samples and percentage area obtained calculating the peak area of each individual trace.

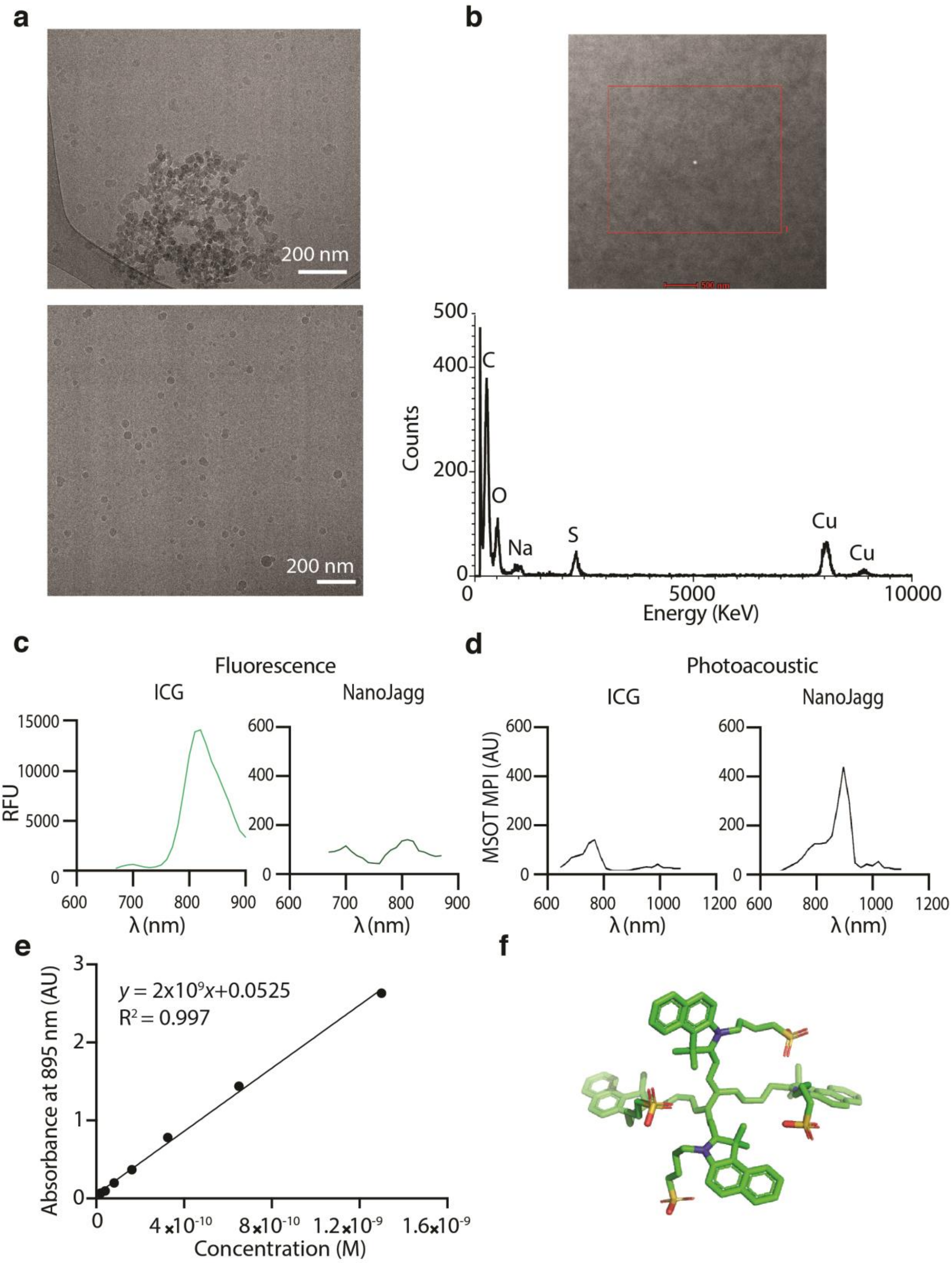

Figure S6. Chemical stability in organic solvents, and photoacoustic properties of ICG and NanoJaggs. (a) Cryo TEM images of NanoJaggs. (b) Energy dispersive X-ray analysis (EDX) demonstrates presence of (C) carbon, (O) oxygen, (Na) sodium, (S) sulfur, and (Cu). (c) Fluorescence emission spectrum excitation at λ_{ex} = 633 nm and (d) photoacoustic spectrum of ICG and NanoJaggs. Both solutions were normalized to a peak absorbance of 1AU prior to analysis. (e) Absorbance vs concentration calibration curve to estimate the extinction coefficient of NanoJagg at the peak absorbance of 895 nm. X-axis (Concentration) is referring to calculated amount of NanoJagg nanoparticles. (f) Structure of the dimer of indocyanine green created in PyMOL.

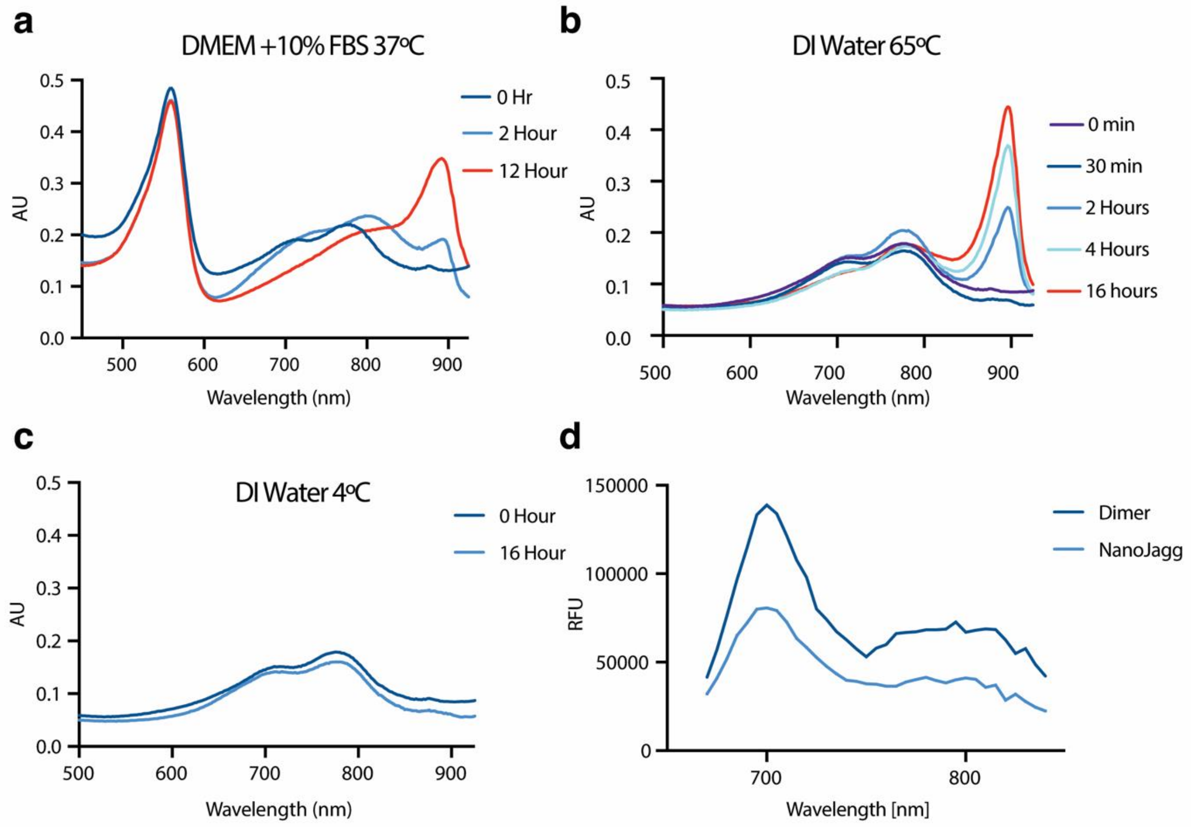

Figure S7. Physiochemical stability of the dimer of ICG in biological media and at different temperatures. (a) ICG dimer in Dulbecco's Modified Eagle Medium (DMEM) + 10% Fetal Bovine Serum (FBS) over time. (b) Dimer of ICG in DI water at 65ºC. (c) Dimer of ICG in DI water at 4ºC. (d) Fluorescent spectrum of the dimer of ICG compared to the NanoJaggs excitation 633nm.

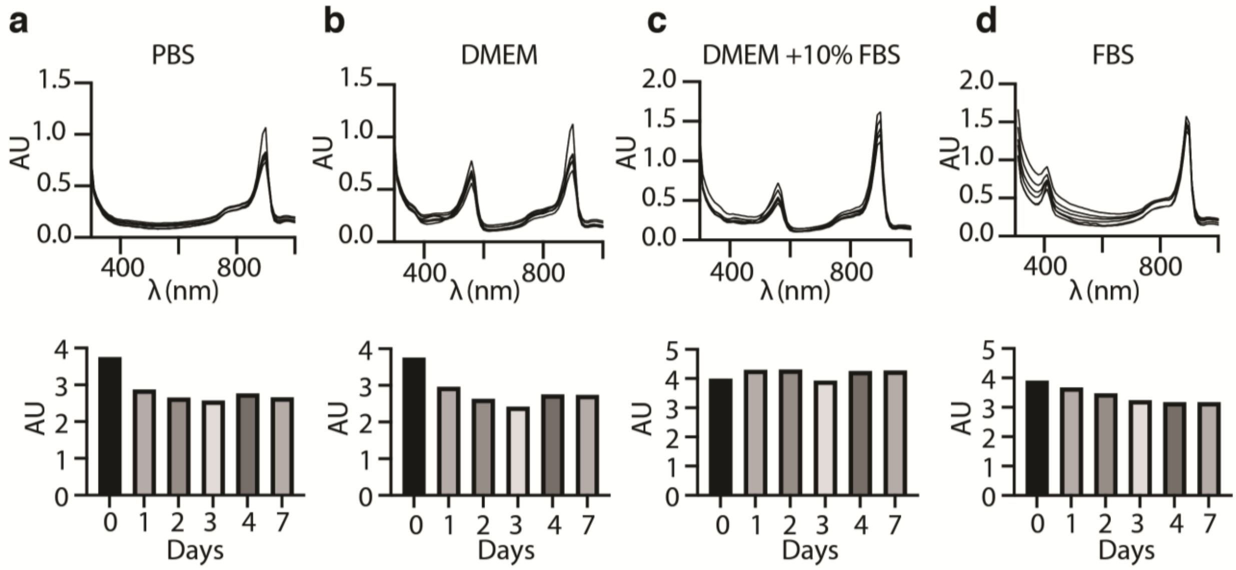

Figure S8. Physiochemical stability of NanoJaggs in biological media. (a) Phosphate buffer pH 7.5, (b) Dulbecco's Modifid Eagle Medium (DMEM), (c) DMEM + 10% Fetal Bovine Serum (FBS) and (d) 100% FBS. NanoJaggs were incubated over 1 week at 37°C and data was obtained by monitoring absorbance spectra at 895nm and 780nm.

5.2. NanoJaggs: *in vitro* evaluation

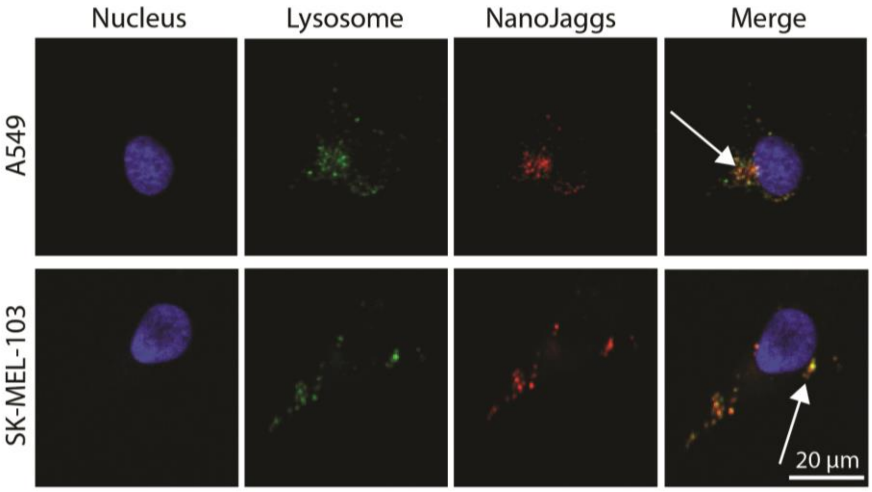

Figure S9. NanoJagg accumulation in the lysosomes of cancer cells. NanoJaggs were added at 10 µg/mL to control lung cancer (A549) and melanoma cancer (SK-MEL-103) cells. Nucleus was stained using Hoechst stain, lysosome with LysoTracker Green, NanoJaggs excited at 633nm and emission between 680-720nm. Confocal images obtained using Zeiss Axio Observer Z1.

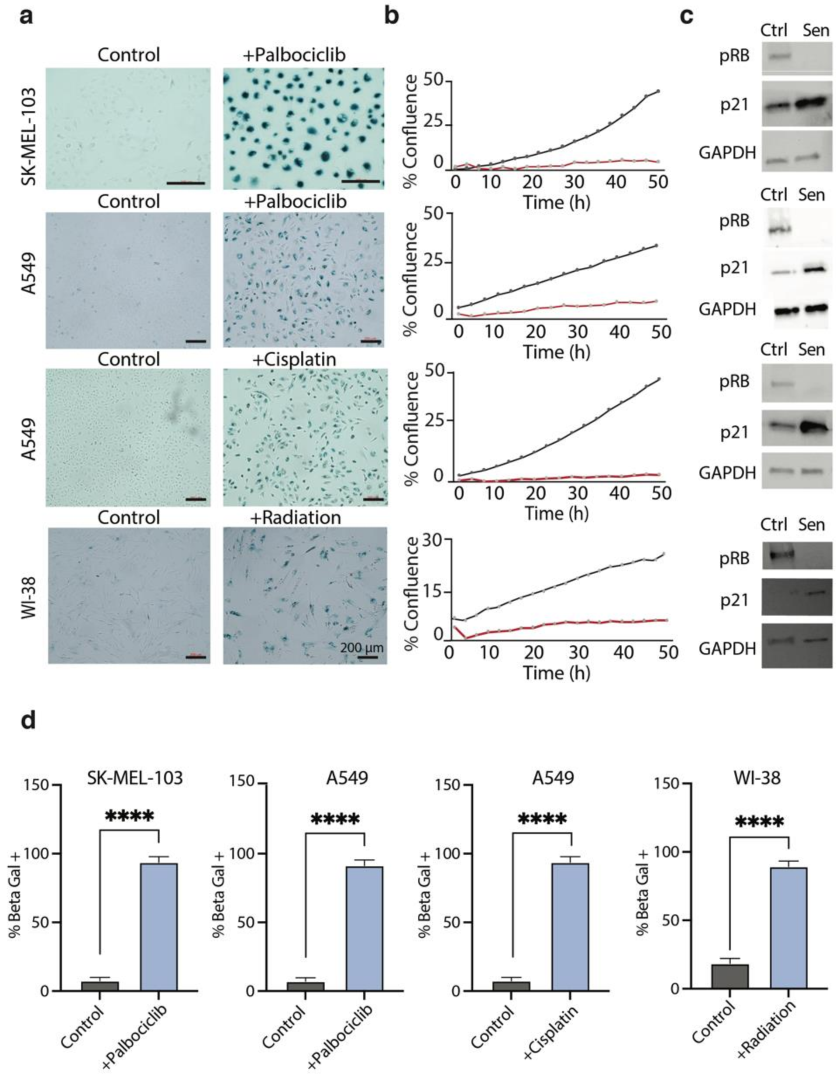

Figure S10. Characterization of senescent cells. Senescent cell is defined as a cell with an increasd expression of β-galactosidase, reduce growth capacity, increased p21 expression and reduced expression of pRB. (a and d) Senescence-associated β-galactosidase staining using a commercial β-galactosidase staining kit. (b) Growth curves of control (black) and senescent (red) cells. (c) Western blots of cellular senescence markers, demonstrating a reduction in pRB, and increased p21 levels in senescent cells compared to controls.

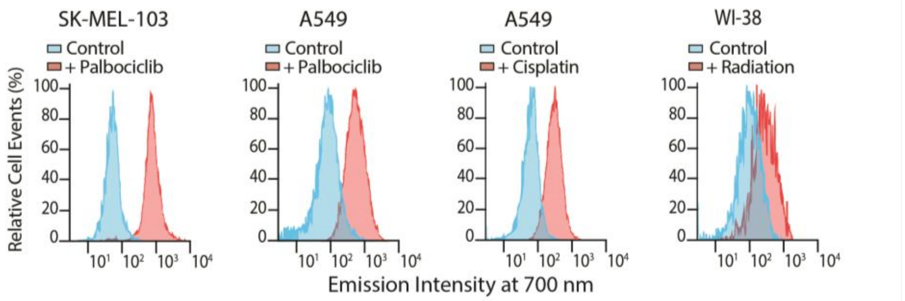

Figure S11. Accumulation of NanoJaggs in control and Senescent cells evaluated with flow cytometry Control (blue) and senescent (red) cells X-axis is log scale and excitation laser of 640 nm was used with a monitored emission of 700 nm.

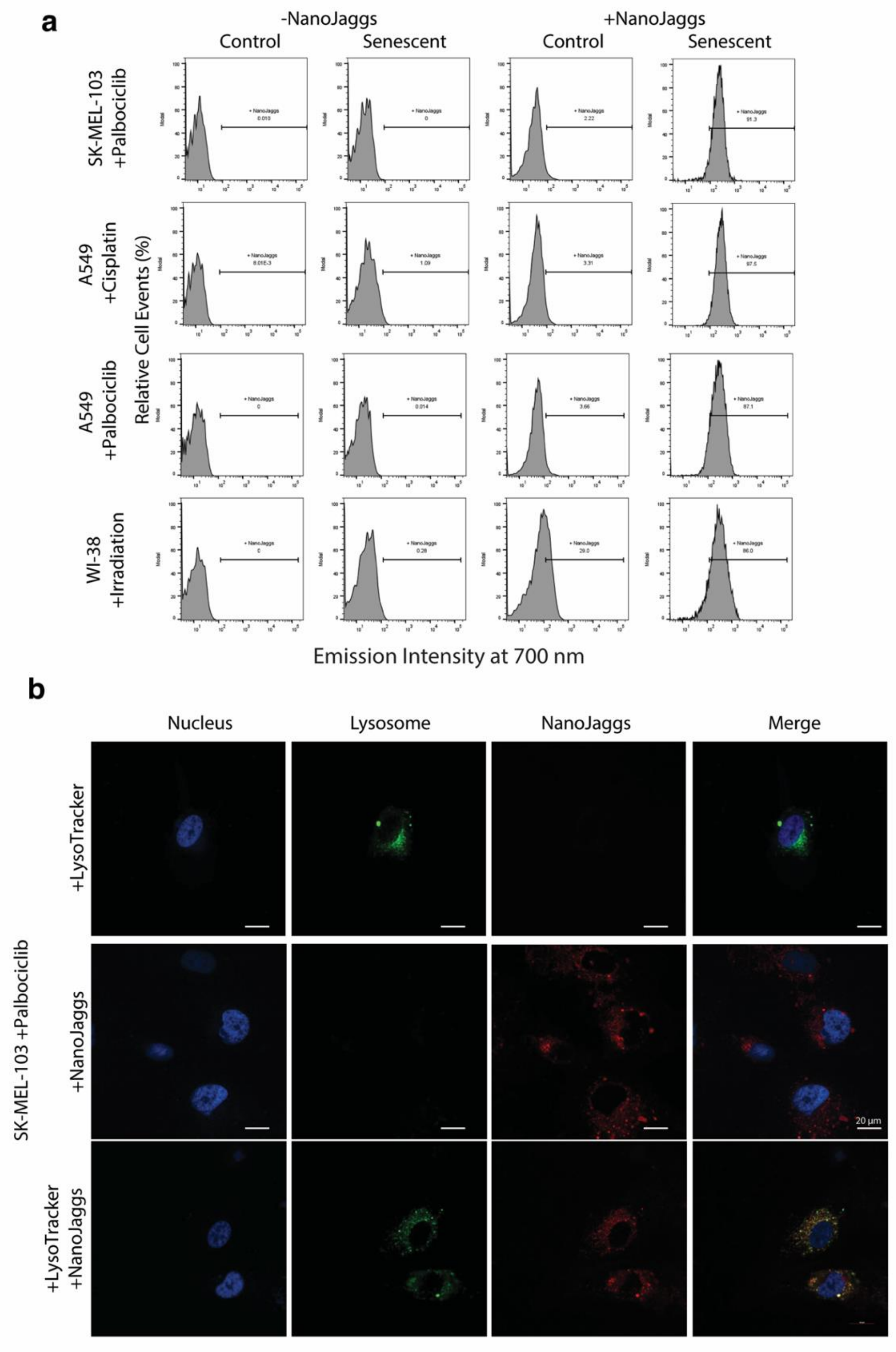

Figure S12. Channel of NanoJaggs is not affected by auto fluorescence, nor does it bleed into other channels. (a) NanoJaggs in control and senescent cells evaluated with flow cytometry. X-axis is log-scale and excitation laser of 640 nm was used with a monitored emission of 700 nm. (b) NanoJaggs signal does not bleed into LysoTracker channel. NanoJaggs excited at 633 nm and emission range 680-720 nm. Confocal images were obtained using Zeiss Axio Observer Z1.

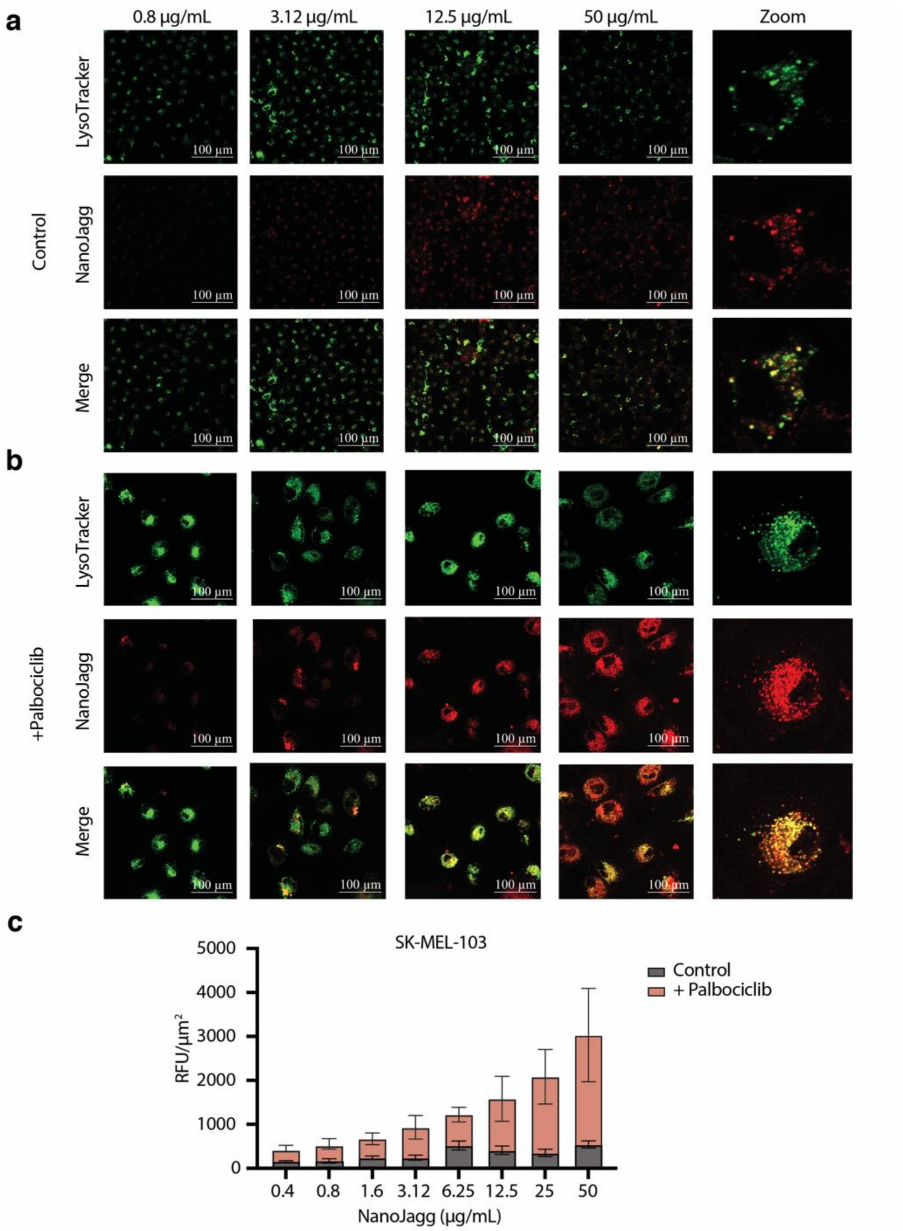

Figure S13. NanoJagg accumulation in the lysosomes of cancer (Control) and senescent (+ Palbociclib) cells. (a) NanoJaggs are taken up by SK-MEL-103 cancer cells and can be found in lysosomes. (b) Senescent SK-MEL-103 cells induced by 7-day treatment with 5 µM of Palbociclib show increased uptake into lysosomes. (c) Quantification of NanoJagg indicate significantly higher accumulation in senescent cells ($p<0.001$). Data represent mean ± SD, and a Two tailed t test was used to calculate the significance (*$p < .05$, **$p < .01$, ***$p < .001$, and ****$p<0.0001$). Lysosome was stained with LysoTracker Green, and NanoJaggs excited at 633 nm and emission range 680-720 nm. Confocal images were obtained using Zeiss Axio Observer Z1.

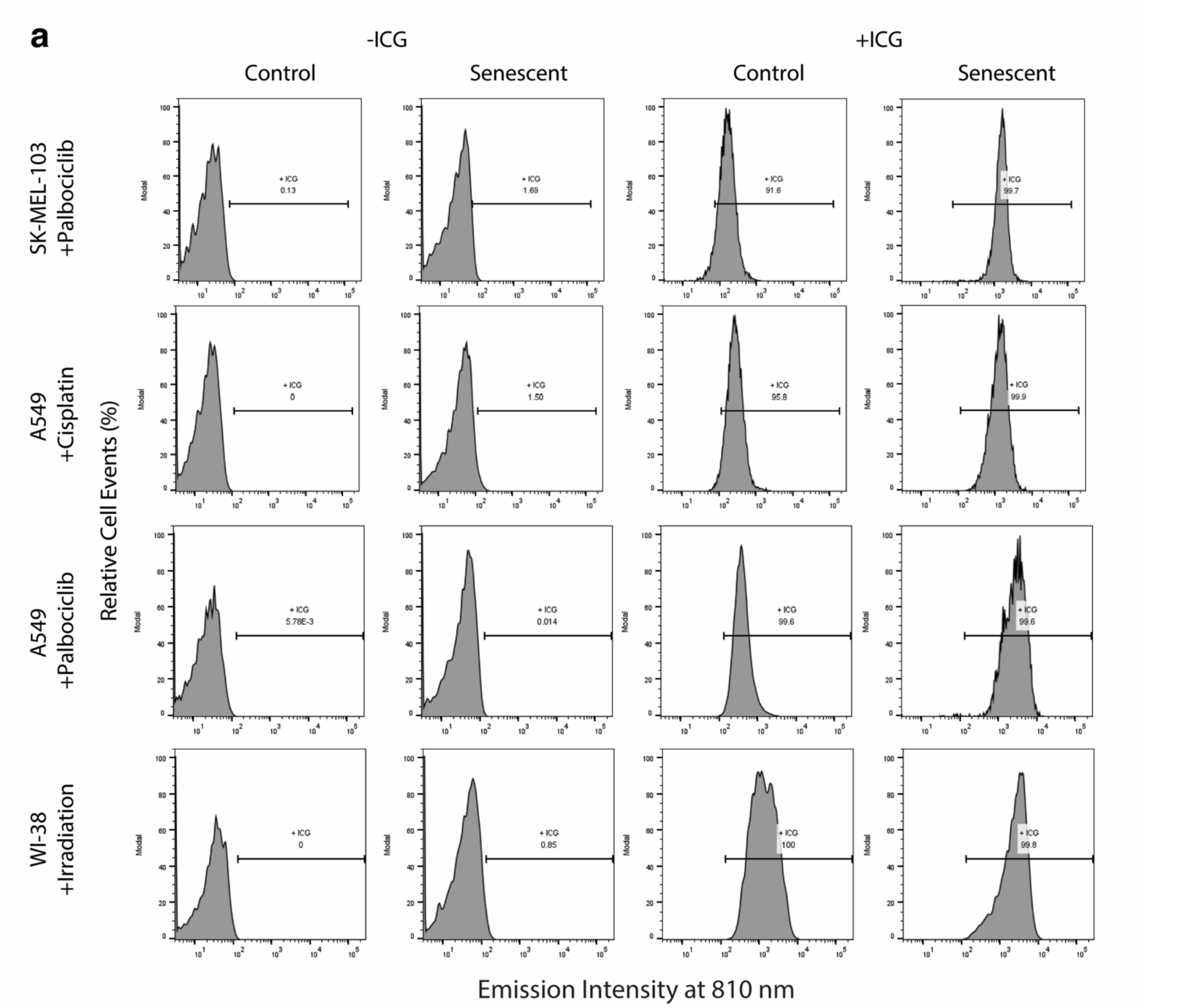

Figure S14. ICG stains both the control and senescent cells. (a) NanoJaggs are taken up by SK-MEL-103 cancer cells and can be found in lysosomes. (b) Accumulation of ICG in control and senescent cells evaluated with flow cytometry control and senescent cells. X-axis is log-scale and excitation laser of 640 nm was used with a monitored emission of 810 nm.

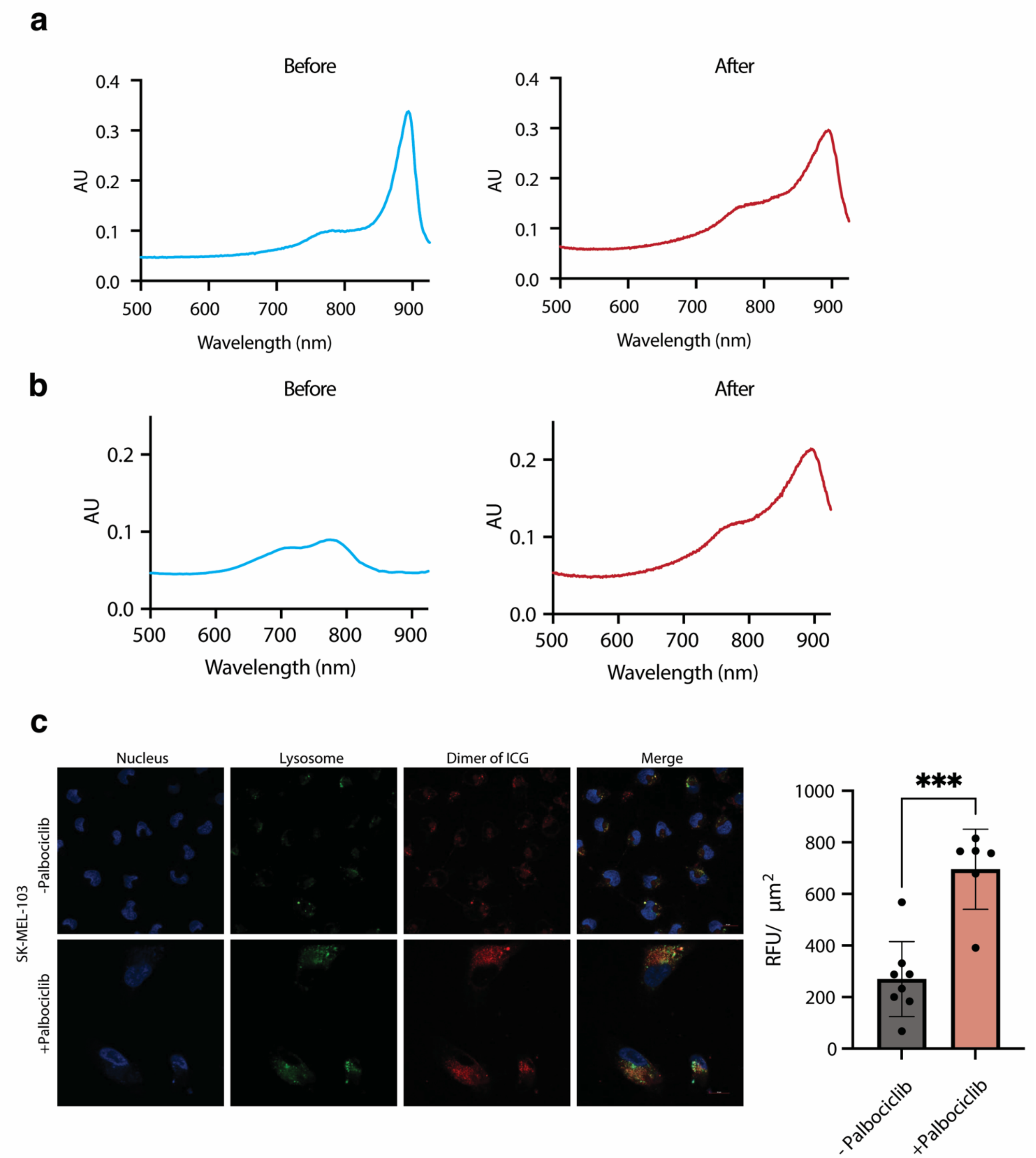

Figure S15. Dimers of ICG rapidly form NanoJaggs over the course of uptake experiments. (a) NanoJaggs remain stable during the course of cell experiments. (b) Dimers of ICG spontaneously form J-aggregates during the course of cell experiments. (c) Accumulation of ICG dimers in control and senescent cells evaluated with confocal microscopy. Data represent mean ± SD, and a Two tailed t test was used to calculate the significance (*p < 0.05, **p < 0.01, ***p < 0.001, and ****p < 0.0001). Lysosome was stained with LysoTracker Green, and NanoJaggs excited at 633 nm and emission range 680-720 nm. Confocal images were obtained using Zeiss Axio Observer Z1.

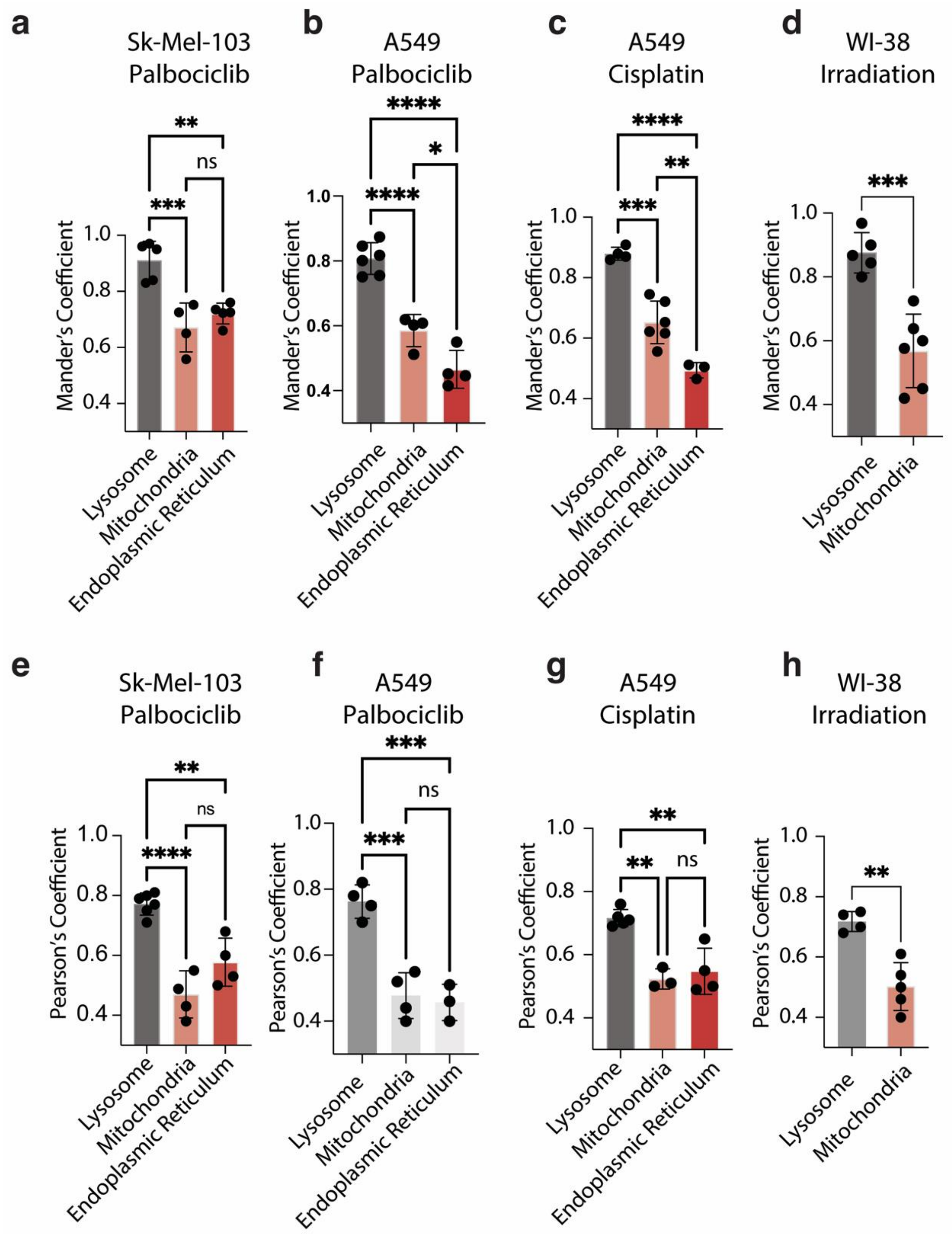

Figure S16. Quantification of NanoJagg colocalization in lysosomes, mitochondria and endoplasmic reticulum using Mander's and Pearson's coefficient. Senescent cells were obtained using different cell treatment: (a and e) senescent SK-MEL-103 cells induced with treatment with 5 µM of Palbociclib (7 days), (b and f) senescent A549 cells induced with 10 µM of Palbociclib (10 days). (c and g) Senescent A549 cells induced with 15 µM of Cisplatin (10 days), and (d and h) senescent Wi-38 cells induced with 10 Gy of X-ray irradiation and then cultured for 10 days. Data is obtained using confocal images, and represent mean ± SD, and a Two tailed t test was used to calculate the significance (*$p < 0.05$, **$p < 0.01$, ***$p < 0.001$, and ****$p < 0.0001$).

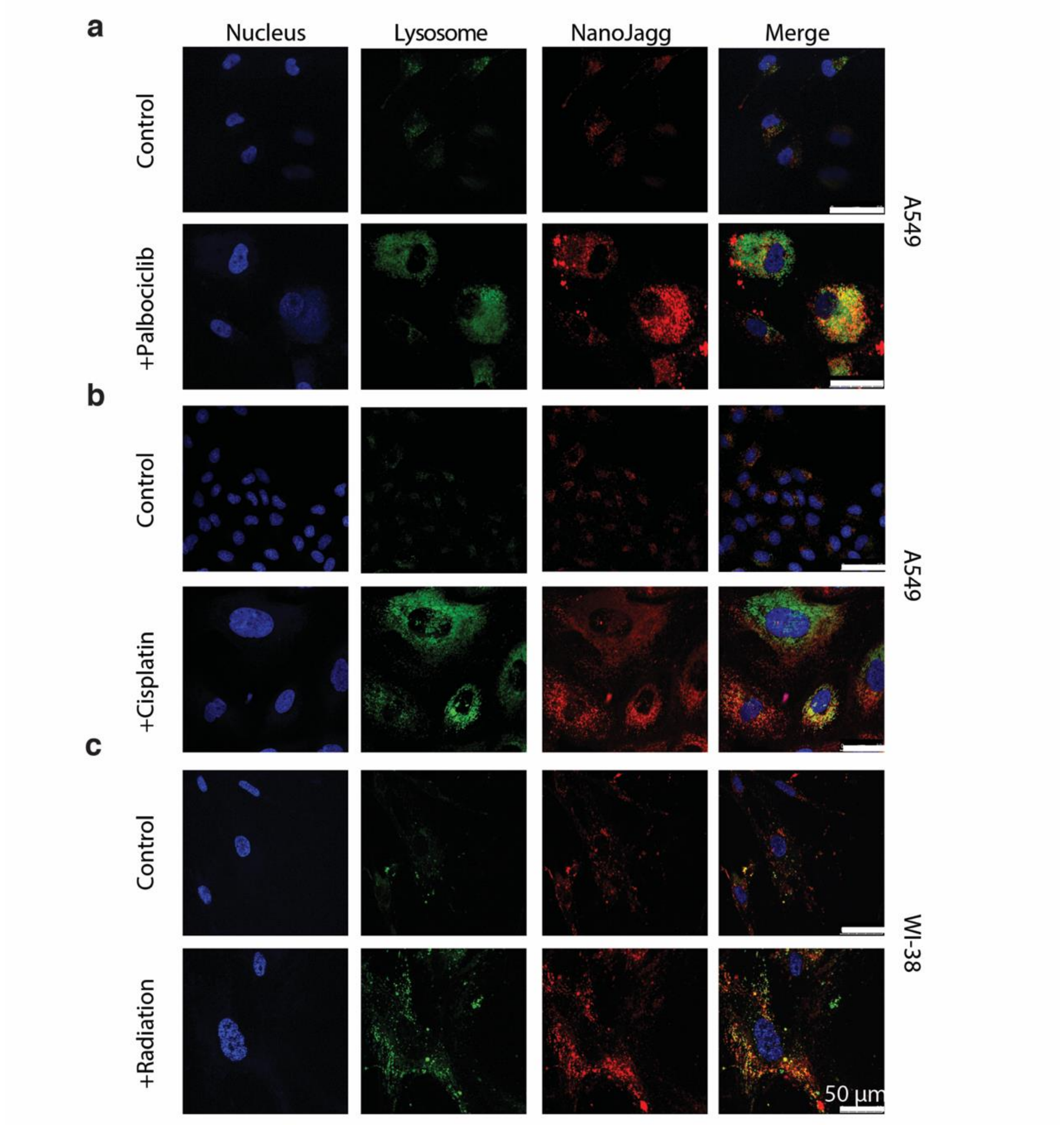

Figure S17. Colocalization of NanoJaggs with the lysosome of senescent cell models. (a) Control and senescent (+Palbociclib) A549 cells. (b) Control and senescent (+Cisplatin) A549 cells. (c) control and senescent (induced by 10 Gy X-ray irradiation) WI-38 cells. Nucleus was stained using Hoechst stain and lysosomes with LysoTracker Green. All samples were treated with 50 µg/mL NanoJaggs and were excited at 633nm and emission range 680-720nm. Confocal images were obtained on a Leica SP5 confocal microscope.

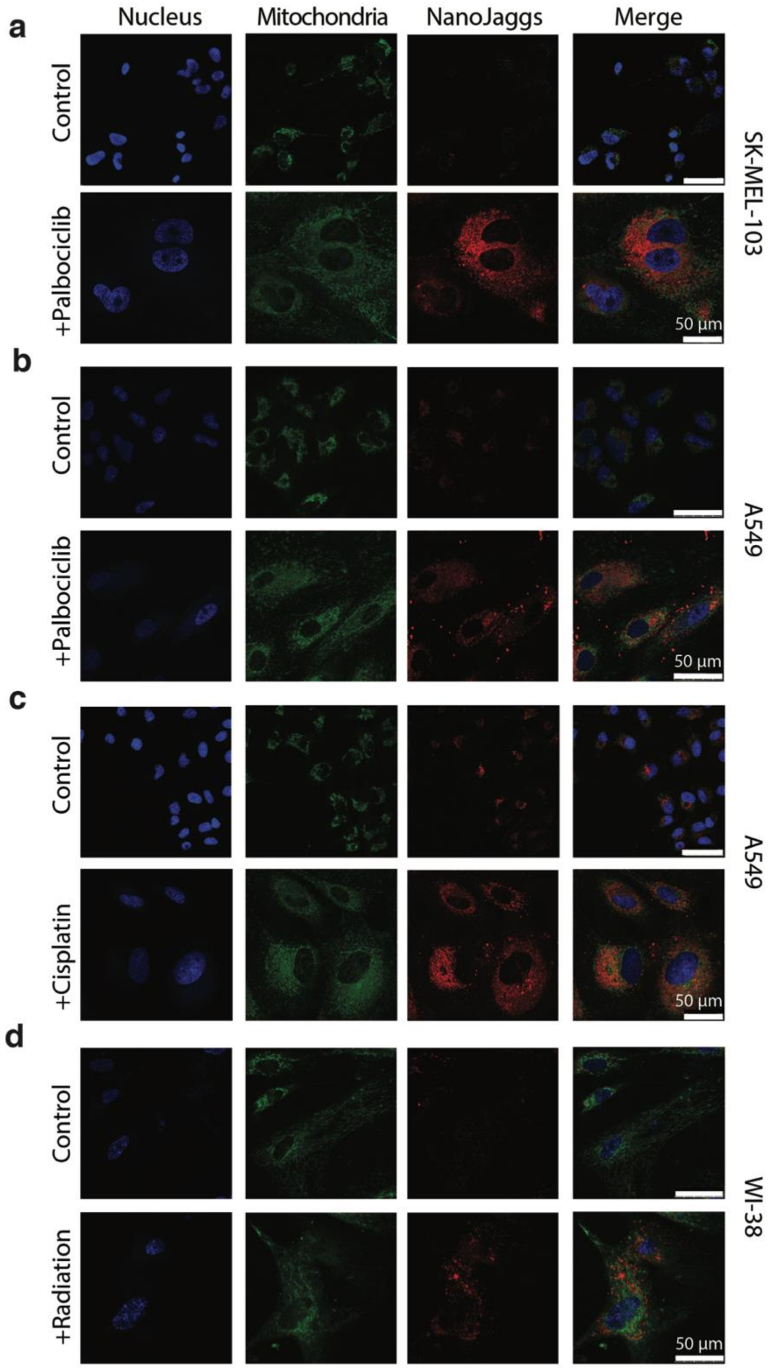

Figure S18. Colocalization of NanoJaggs with mitochondria. (a) SK-MEL-103k control and senescent cells (+Palbociclib). (b) A549 control and senescent cells (+Palbociclib). (c) A549 control and senescent cells (+Cisplatin,CDDP). (d) WI-38 control and senescent cells induced by X-ray irradiation. Nucleus was stained using Hoechst stain, mitochondria with MitoTracker Green. All samples were treated with 50 µg/mL NanoJaggs and were excited at 633nm and emission range 680-720nm. Confocal images were obtained on a Leica SP5 confocal microscope.

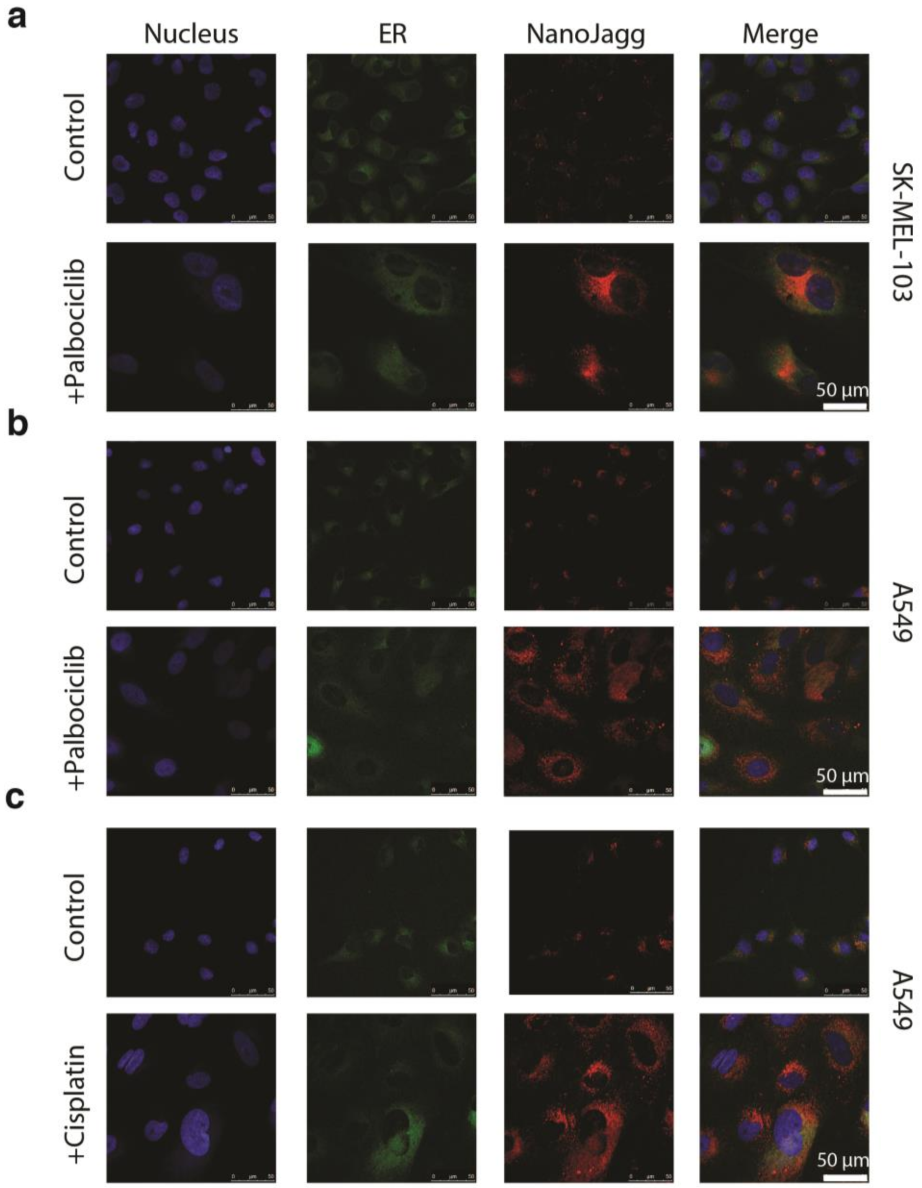

Figure S19. Colocalization of NanoJaggs with the endoplasmic reticulum (a) SK-MELel-103 control and senescent cells (+Palbociclib). (b) A549 control and senescent cells (+ Palbociclib) and (c) A549 control and senescent cells (+cisplatin, CDDP). Nucleus was stained using Hoechst stain, and endoplasmic reticulum with ER Tracker Green. All samples were treated with 50 µg/mL NanoJaggs and were excited at 633nm and emission range 680-720nm. Confocal images were obtained on a Leica SP5 confocal microscope.

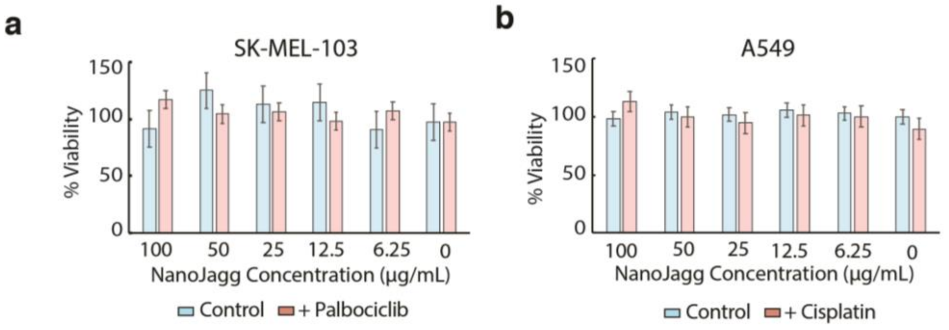

Figure S20. Viability of control and senescent cells treated with different concentrations of NanoJaggs. (a) Senescence of SK-MEL-103 was induced by 5 µM Palbociclib (7 days) and (b) Senescence of A549 cells was induced by 15 µM Cisplatin (10 days). Data represent mean ± SD of N=3 biological repeats. Cell viability was assessed using the CellTiter-Blue assay.

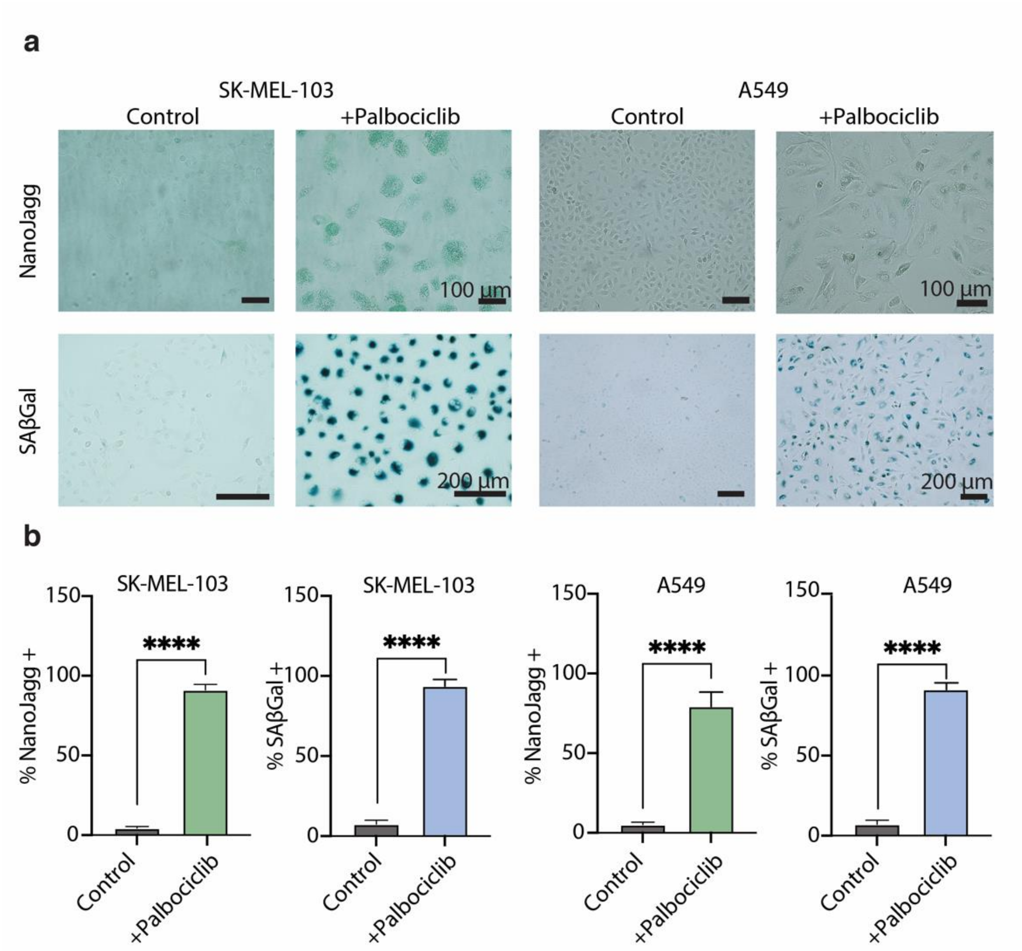

Figure S21. Comparison of commercial X-gal (SAβ-galactosidase) stain for senescent cells and NanoJaggs. (a) Control and senescent SK-MEL-103 and A549 (both +Palbociclib) cells stained with NanoJaggs and X-gal dye. (b) ~90% of treated SK-MEL-103 cells were positive for NanoJaggs, and ~95% were positive for SA-β-Gal. ~80% of treated A459 cells were positive for NanoJaggs and ~95% are positive to SA-β-Gal. Cells were treated with 50 µg/mL of NanoJaggs and counted to calculate % NanoJagg+ cells.

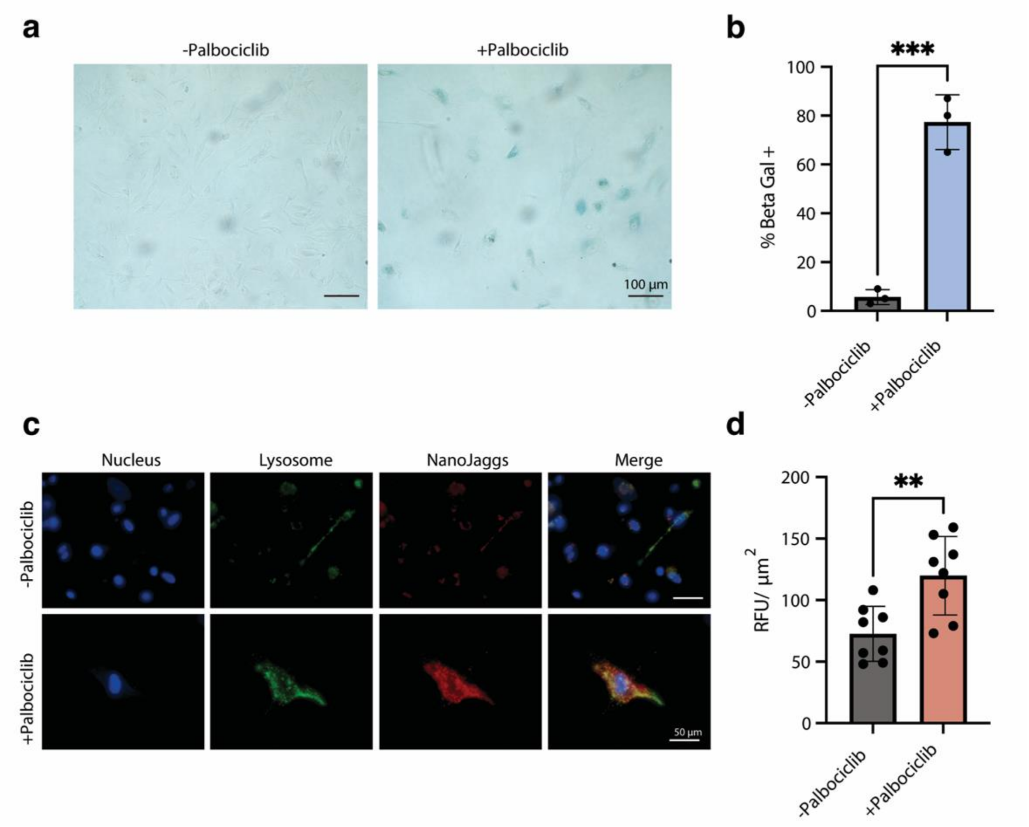

Figure S22. NanoJagg accumulation in human umbilical vein endothelial cells (HUVECs) control and senescent (+ Palbociclib) cells. (a) images of SA-β-Gal staining on HUVECs both treated and untreated with palbociclib. Senescent HUVECs were induced by 7-day treatment with 2 µM of Palbociclib. (b) Quantification of % positive SA-β-Gal cells after palbociclib treatment. (c) Confocal images of NanoJaggs in HUVECs both treated and untreated with Palbociclib. (d) Quantification of NanoJagg indicate significantly higher accumulation in senescent cells ($p < 0.01$). Data represent mean ± SD, and a Two tailed t test was used to calculate the significance (*$p < 0.05$, **$p < 0.01$, ***$p < 0.001$, and ****$p < 0.0001$). Lysosome was stained with LysoTracker Green, and NanoJaggs excited at 633 nm and emission range 680-720 nm. Confocal images were obtained using Zeiss Axio Observer Z1.

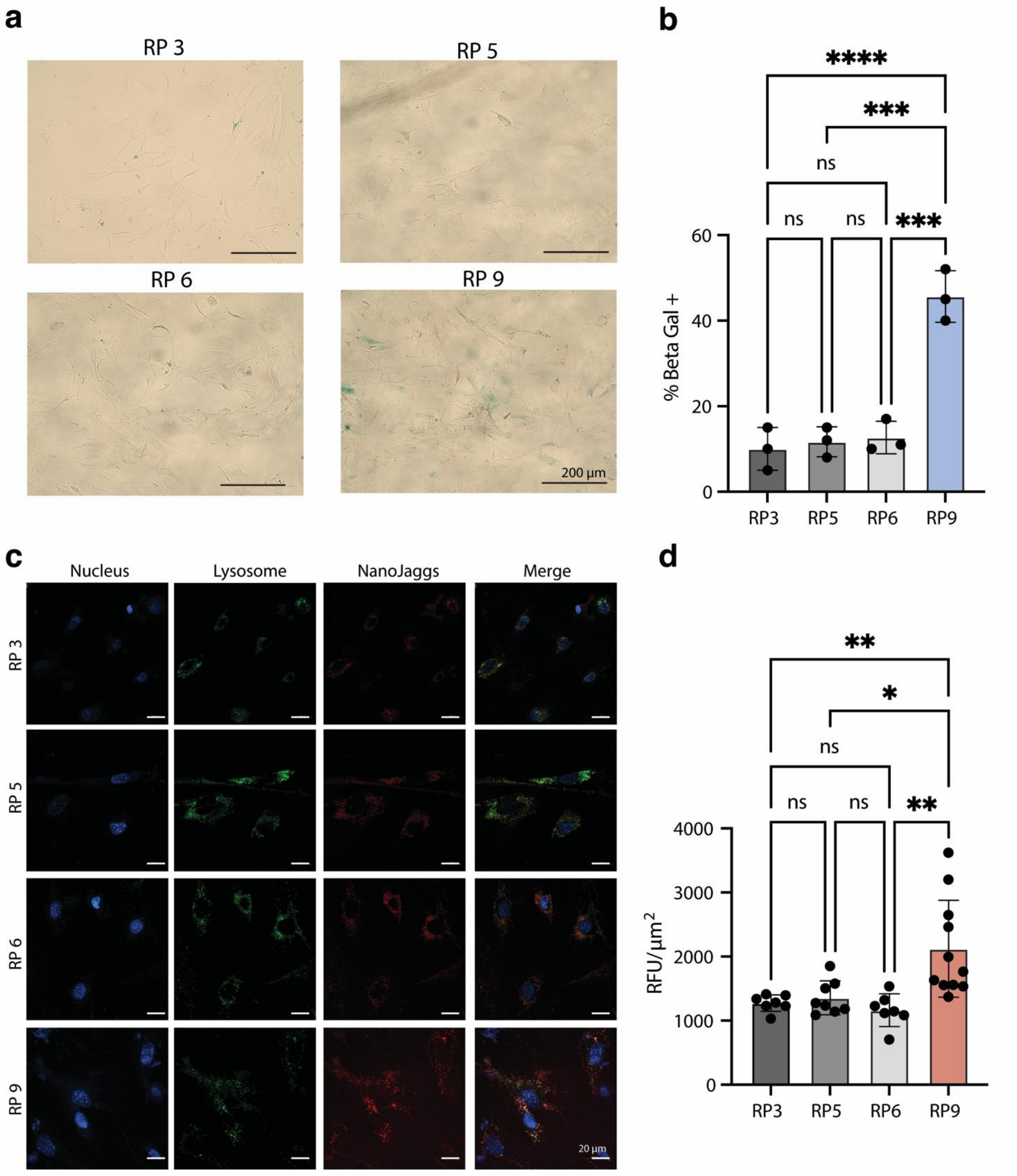

Figure S23. NanoJagg accumulation in mouse embroyonic fibroblasts (MEFs) induced with replicative exhaustion. (a) Representative images of SA-β-Gal staining over replicative passage number. (b) Quantification of % positive SA-β-Gal cells after replicative exhaustion, over passages 3 to 9. (c) Confocal images of MEFs incubated with NanoJaggs. Nucleus was stained using Hoechst stain, lysosome with LysoTracker Green, NanoJaggs excited at 633nm and emission between 680-720 nm. (d) Quantification of NanoJagg signal over replicative passages ($p<.01$). Data represent mean ± SD, and a Two tailed t test was used to calculate the significance (*$p < 0.05$, **$p < 0.01$, ***$p < 0.001$, and ****$p < 0.0001$). Confocal images obtained using Zeiss Axio Observer Z1.

5.3. NanoJaggs: Endocytosis studies

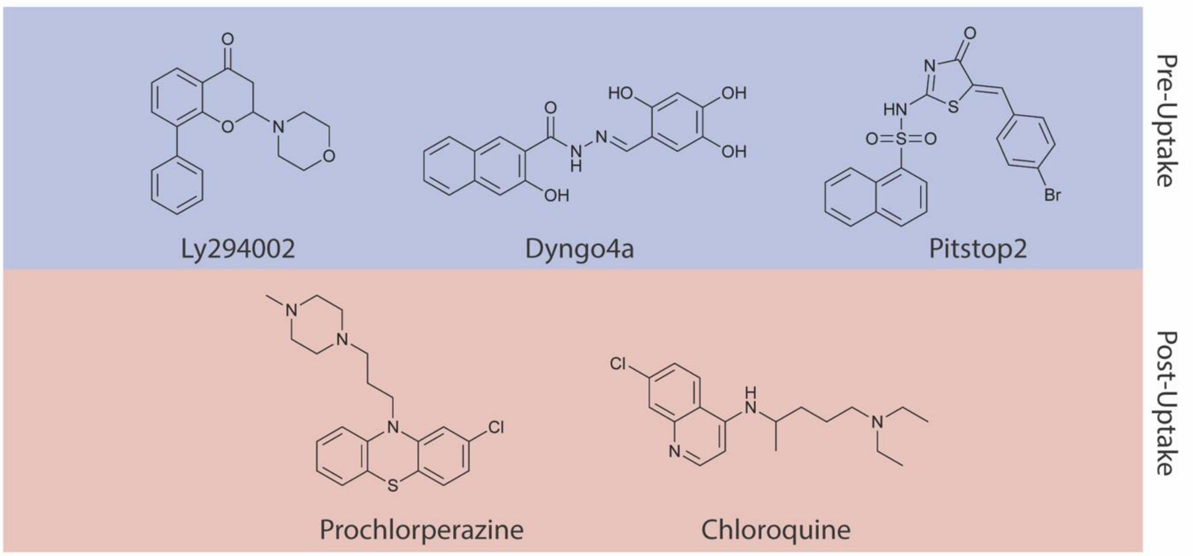

Figure S24. Pre-uptake and post-uptake inhibitors employed in the study**.** The structures of pre-uptake inhibitor Ly294002 (inhibits micropinocytosis), Dyngo4a (inhibits dynamin driven processes), and Pistop2 (inhibits the formation of clathrin coated pits.) are shown in the upper panel. Lower panel shows the structures of the post-uptake inhibitors Prochlorperazine (PCZ, inhibitor of clathrin coated pit's fission), and chloroquine (inhibitor of autophagy).

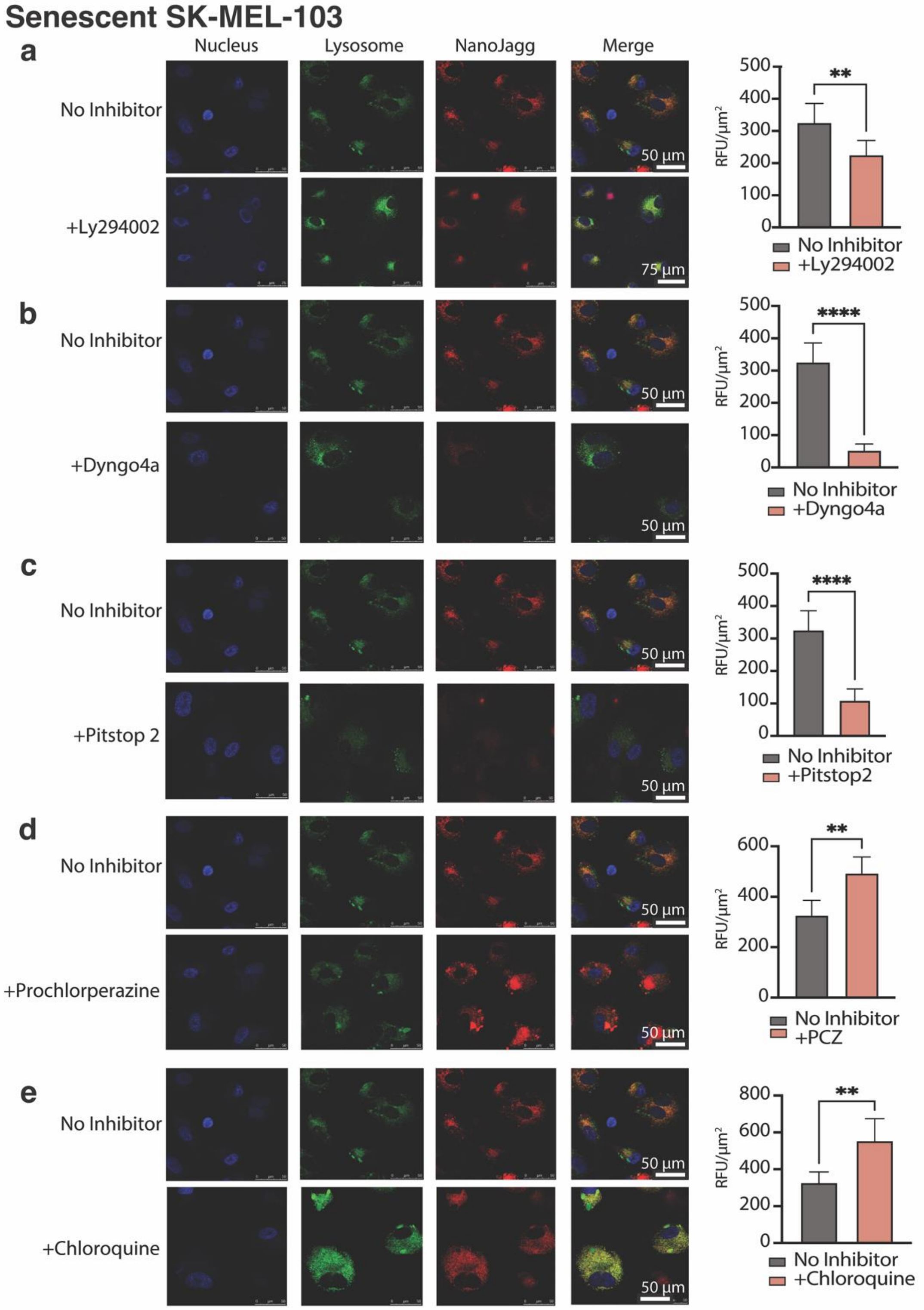

Figure S25. Colocalization of NanoJaggs in senescent SK-MEL-103 cells in presence of different inhibitors. Cells were treated with NanoJaggs in presence and absence of inhibitors: (a) LY294002 (20–50 µM, (b) Dyngo4a (10–30 µM), (c) Pitstop2 (5–15 µM), (d) Prochlorperazine dimaleate salt (15 µM) and (e) Chloroquine (50 µM). Nucleus was stained using Hoechst stain, and lysosome with LysoTracker Green. All samples were treated with 50 µg/mL NanoJaggs and were excited at 633nm and emission range 680-720nm. Confocal images were obtained on a Leica SP5 confocal microscope. Data represent mean ± SD, obtained from 3 biological replicates (N=3). Two tailed t test was used to calculate the significance (*p < 0.05, **p < 0.01, ***p < 0.001, and ****p < 0.0001).

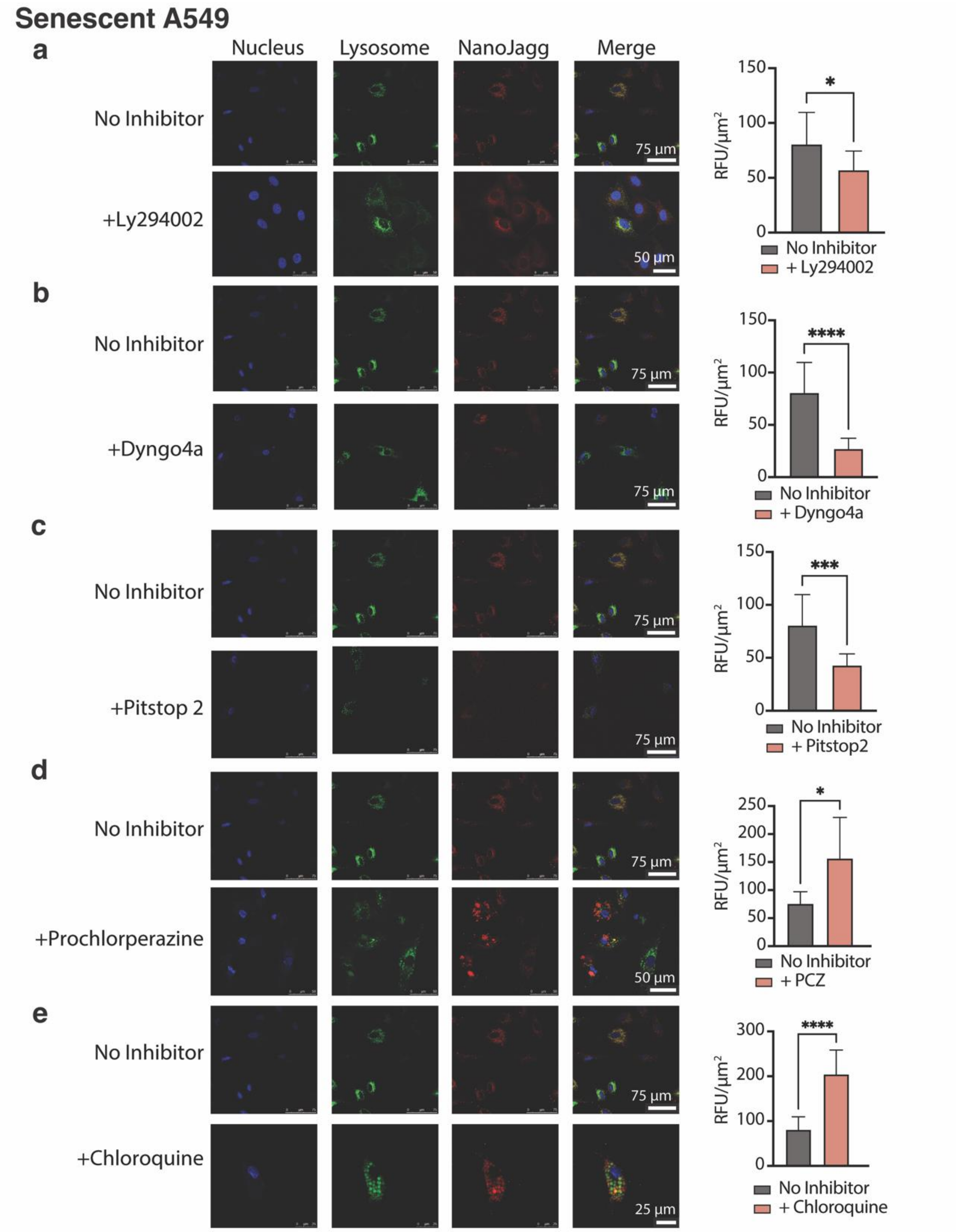

Figure S26. Colocalization of NanoJaggs in senescent A459 cells in presence of different inhibitors. Cells were treated with NanoJaggs in presence and absence of inhibitors: (a) LY294002 (20–50 µM, (b) Dyngo4a (10–30 µM), (c) Pitstop2 (5–15 µM), (d) Prochlorperazine dimaleate salt (15 µM) and € Chloroquine (50 µM). Nucleus was stained using Hoechst stain, and lysosome with LysoTracker Green. All samples were treated with 50 µg/mL NanoJaggs and were excited at 633nm and emission range 680-720nm. Confocal images were obtained on a Leica SP5 confocal microscope. Data represent mean ± SD, obtained from 3 biological replicates (N=3). Two tailed t test was used to calculate the significance (*p < 0.05, **p < 0.01, ***p < 0.001, and ****p < 0.0001).

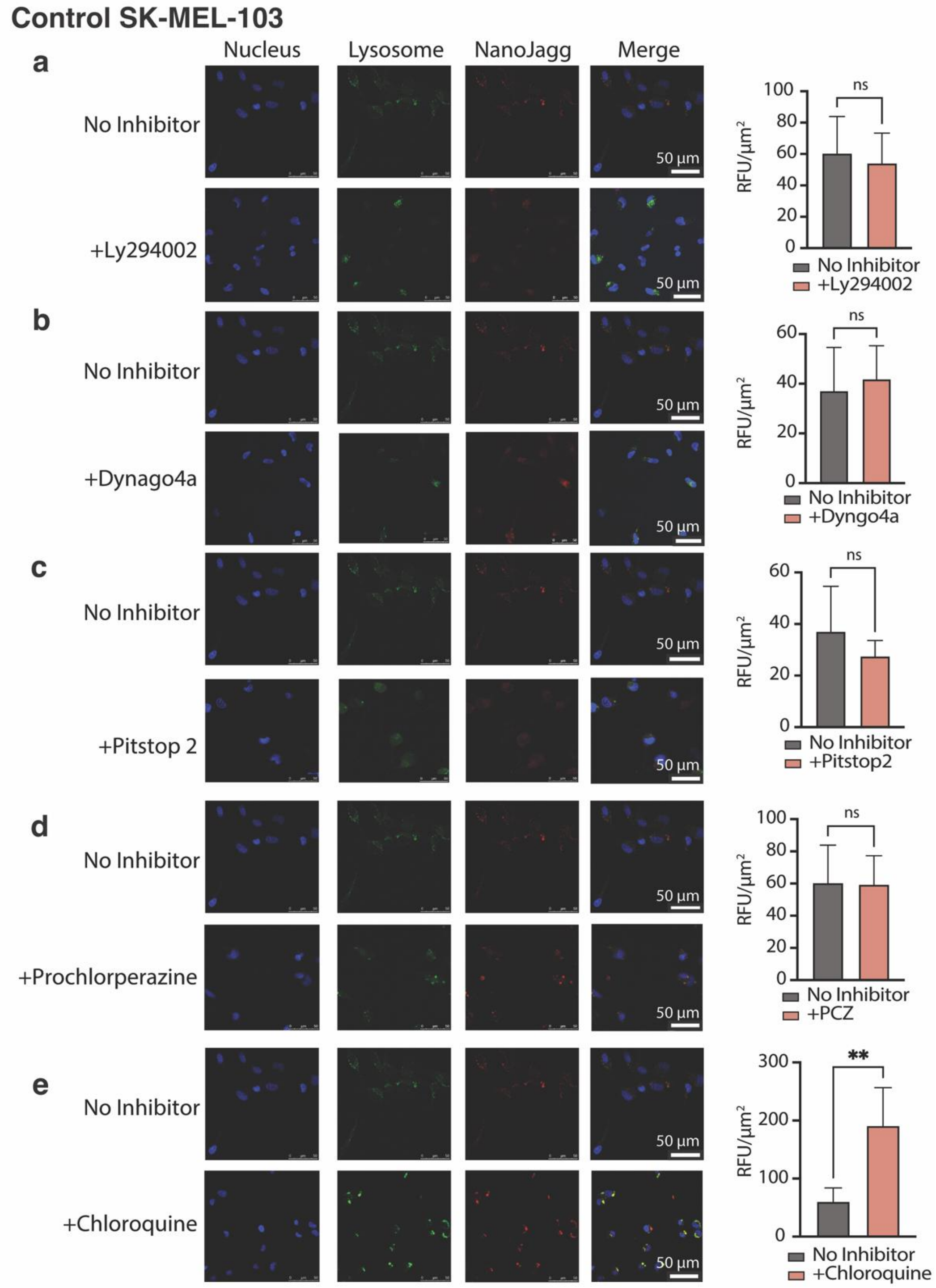

Figure S27. Colocalization of NanoJaggs in non-senescent SK-MEL-103 control cells in presence of inhibitors. Cells were treated with NanoJaggs in presence and absence of inhibitors: (a) LY294002 (20–50 µM, (b) Dyngo4a (10–30 µM), (c) Pitstop2 (5–15 µM), (d) Prochlorperazine dimaleate salt (15 µM) and (e) Chloroquine (50 µM Nucleus was stained using Hoechst stain, and lysosome with LysoTracker Green. All samples were treated with 50 µg/mL NanoJaggs and were excited at 633nm and emission range 680-720nm. Confocal images were obtained on a Leica SP5 confocal microscope. Data represent mean ± SD, obtained from 3 biological replicates (N=3). Two tailed t test was used to calculate the significance (*p < 0.05, **p < 0.01, ***p < 0.001, and ****p < 0.0001).

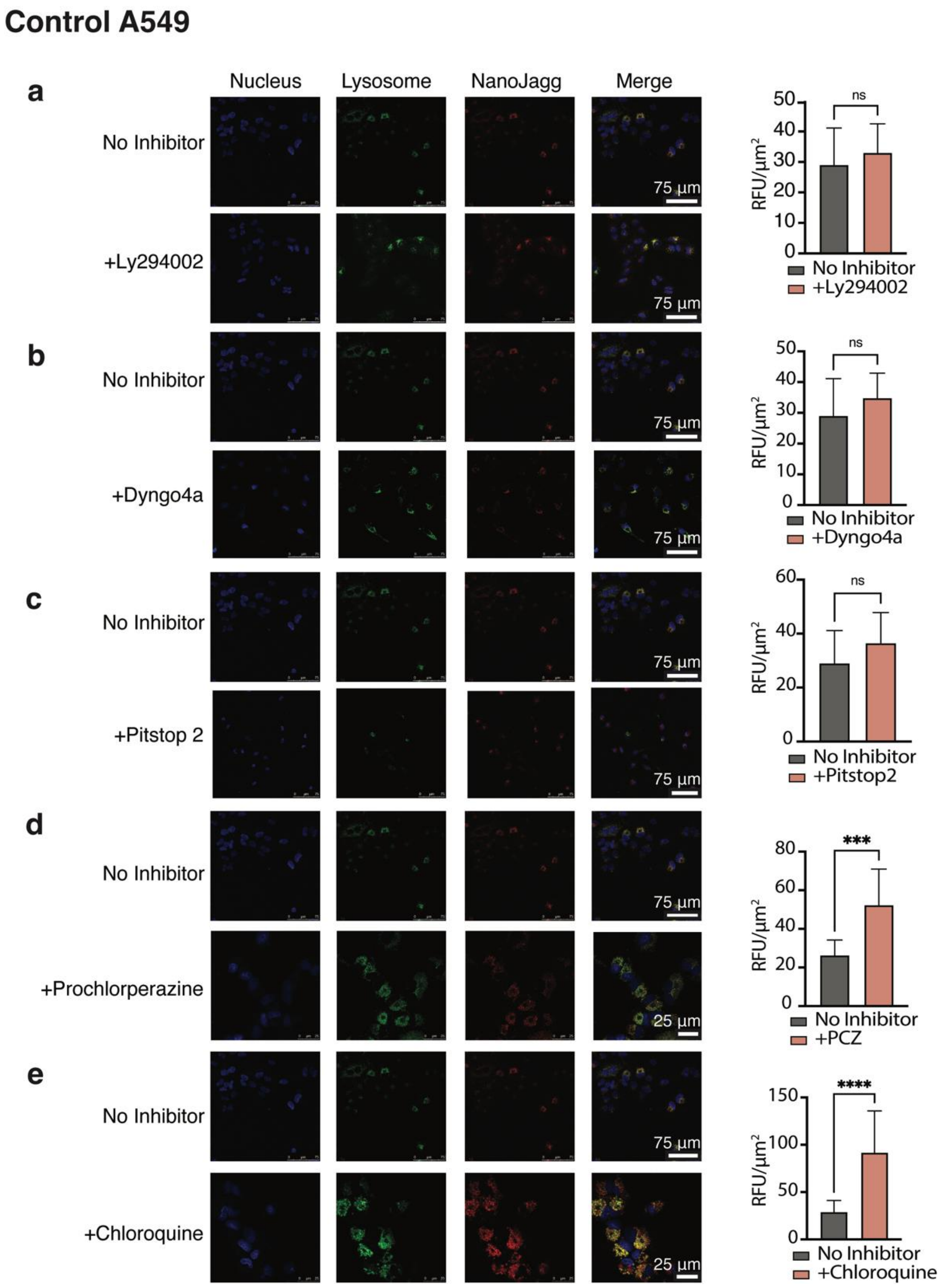

Figure S28. Colocalization of NanoJaggs in non-senescent A459 control cells in presence of inhibitors. Cells were treated with NanoJaggs in presence and absence of inhibitors: (a) LY294002 (20–50 µM, (b) Dyngo4a (10–30 µM), (c) Pitstop2 (5–15 µM), (d) Prochlorperazine dimaleate salt (15 µM) and (e) Chloroquine (50 µM). ($p<0.01$). Nucleus was stained using Hoechst stain, and lysosome with LysoTracker Green. All samples were treated with 50 µg/mL NanoJaggs and were excited at 633nm and emission range 680-720nm. Confocal images were obtained on a Leica SP5 confocal microscope. Data represent mean ± SD, obtained from 3 biological replicates (N=3). Two tailed t test was used to calculate the significance (*$p < .05$, **$p < 0.01$, ***$p < 0.001$, and ****$p < 0.0001$).

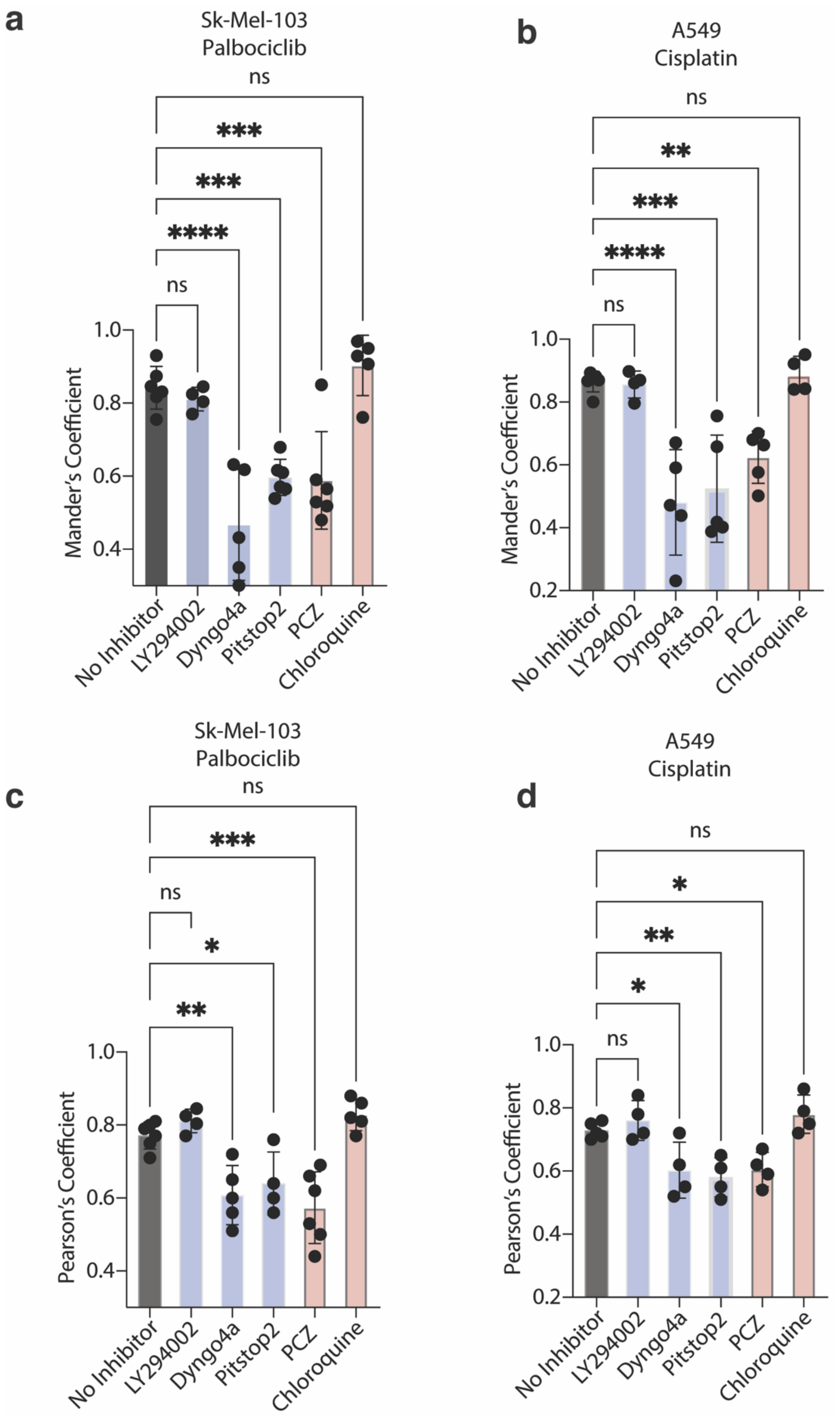

Figure S29. Colocalization of NanoJaggs in lysosomes of senescent cells in presence of inhibitors using Mander's and Pearson's coefficient. Senescent SK-MEL-103 cells induced with 5 µM of Palbociclib for 7 days (a and c) and senescent A549 cells induced with 15 µM of Cisplatin for 10 days (b and d). Data was obtained from confocal images from Fig. S18 and S19 comparing the overlap of the lysosome and NanoJagg channel. Data represent mean ± SD, and a Two tailed t test was used to calculate the significance, the Bonferroni correction was applied (*p < 0.05, **p < 0.01, ***p < 0.001, and ****p < 0.0001).

5.4. NanoJagg *ex vivo* evaluation

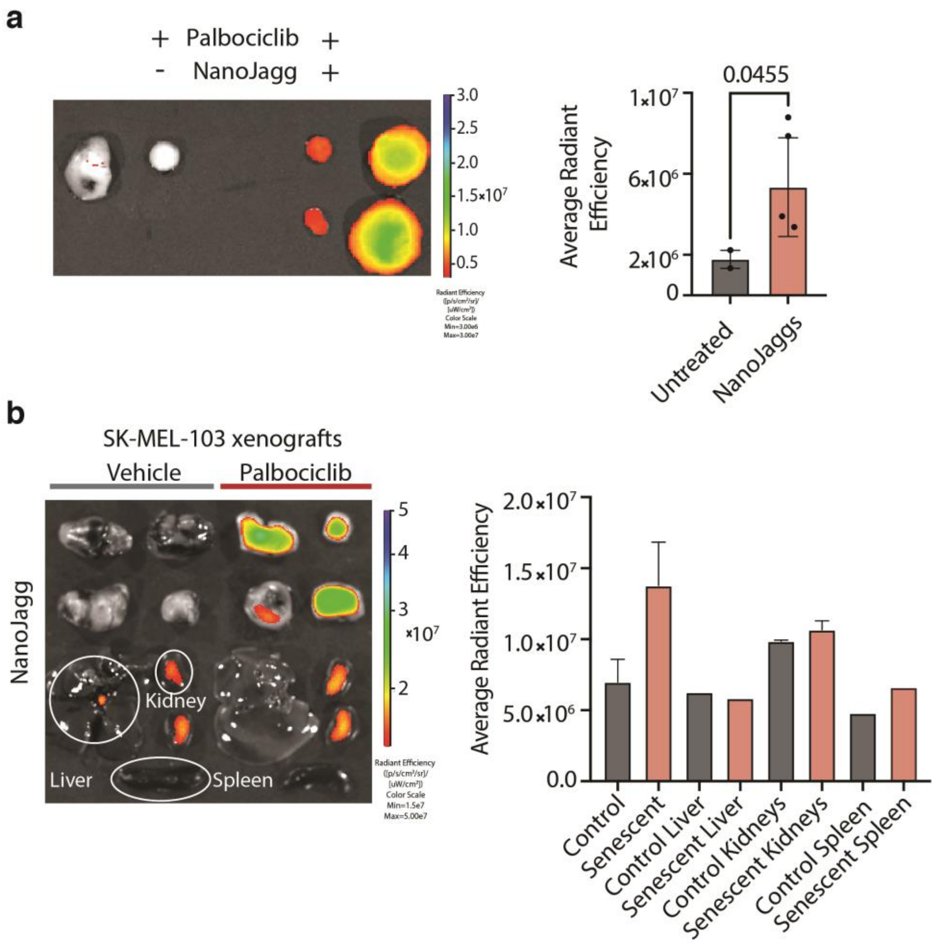

Figure S30. Post-in vivo assessment of NanoJaggs distribution in different organs. (a) Image of tumors and one set of organs from both untreated (non-senescent tumor) and Palbociclib-treated (senescent tumor) mice. (b) NanoJaggs signal in untreated and Palbociclib-treated tumors and in organs. Excitation at 610nm and emission at 700-720nm was used to monitor NanoJaggs signal using an IVIS Spectrum Imaging System. Significant NanoJaggs presence was detected in kidneys of both control and senescent mice indicating renal clearance. Data in graphs represent mean of the average radiant efficiency ± SD quantified from images.

5.5. NanoJagg *in vivo* evaluation with fluorescent imaging

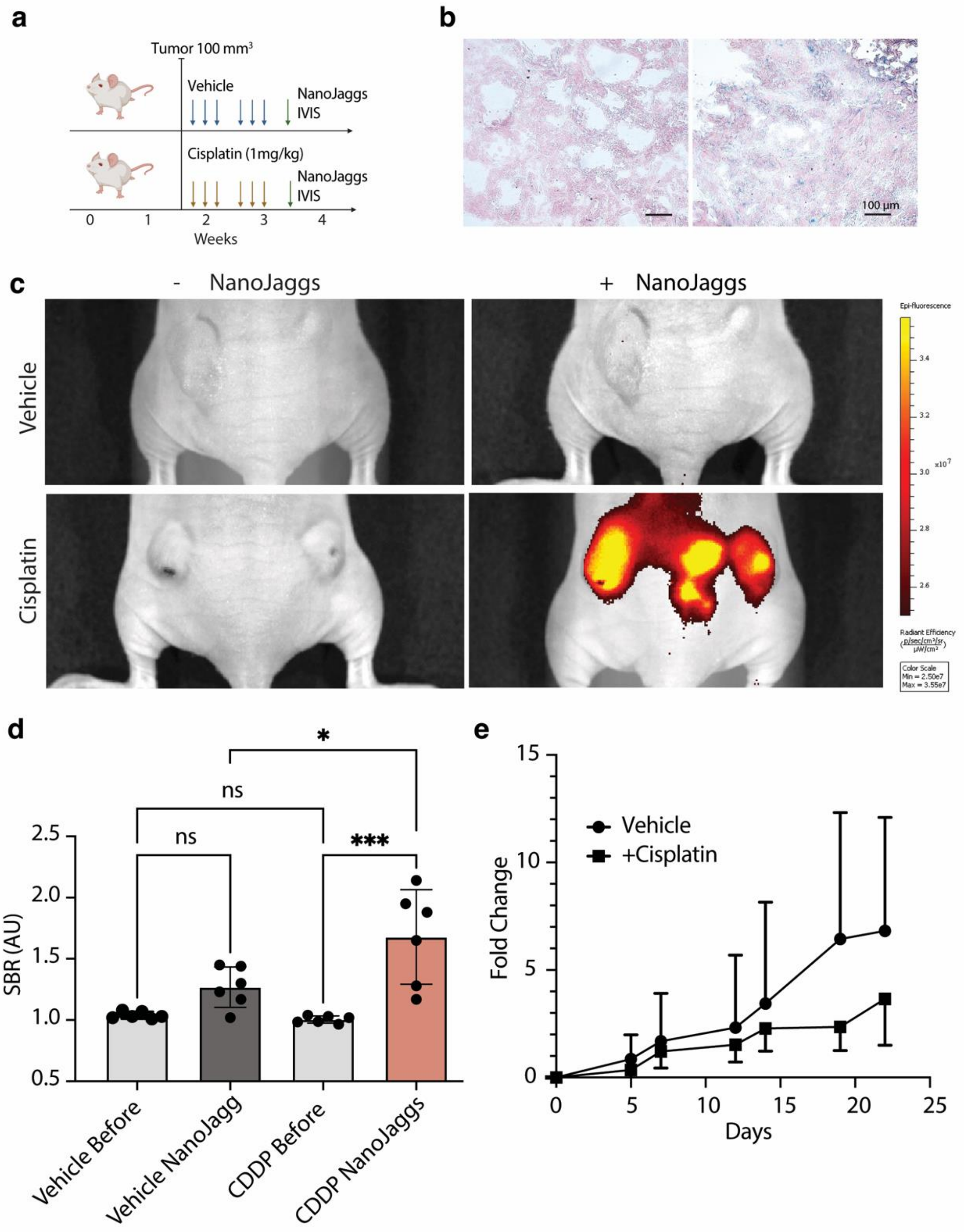

Figure S31. *In vivo* assessment of NanoJaggs in A549 xenograft model using fluorescence imaging. (a) Schematic overview of the *in vivo* experiment, the uptake the NanoJaggs was compared in Cisplatin treated tumors *vs* untreated tumors. (b) SA-β-Gal staining of untreated and Cisplatin treated tumors. (c) IVIS fluorescence images of vehicle and treated mice before (left) and after (right) treatment with NanoJaggs. (d) Quantification of the signal to background ratio of NanoJagg fluorescent signal ($p < 0.001$) compared to before, and ($p < 0.05$) compared to the vehicle. (e) Growth curve of tumors from mice treated with vehicle and Cisplatin. Data represent mean ± SD, and a Two-tailed t-test was used to calculate the significance. (*$p < 0.05$, **$p < 0.01$, ***$p < 0.001$).

5.6. NanoJagg *in vivo* evaluation with photoacoustic imaging

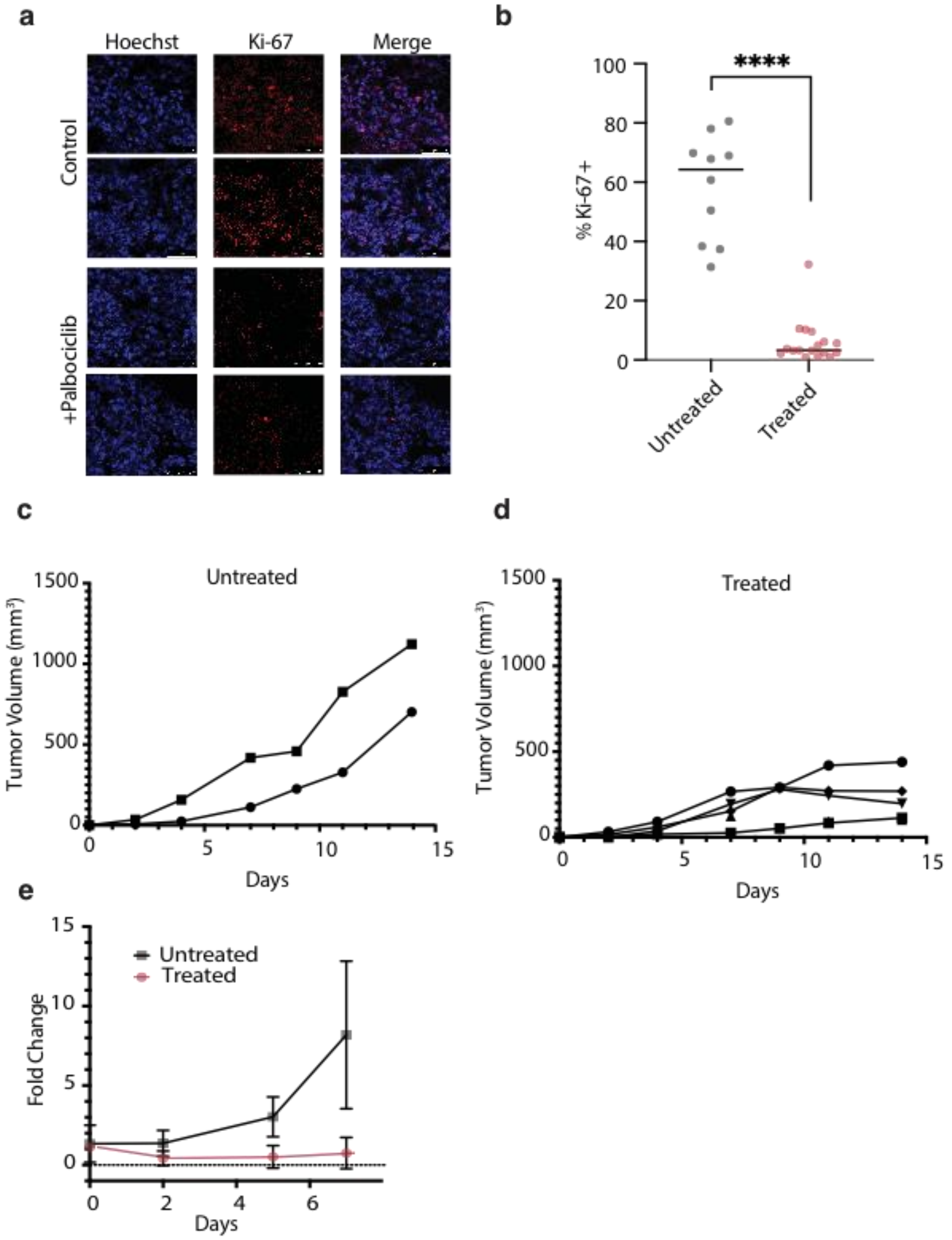

Figure S32. Characterization of tumor senescence using standard senescence markers. (a) Ki-67 staining with immunofluorescence. Treated tumors were compared to untreated and multiple slices and areas were used. (b) Quantification data obtained from confocal images ($p < 0.0001$). Growth curves of the untreated and treated tumors indicating the proliferation of the untreated tumor and the growth arrest of treated (senescent) tumor (c and d). (e) Fold change representation of the data from b and c to highlight the growth arrest in treated tumors and compare it to the untreated tumors. Palbociclib treated (senescent) tumors show a marked decrease in proliferation rate indicative of senescence induction. Two tailed t test was used to calculate the significance (*p < 0.05, **p < 0.01, ***p < 0.001, and ****p < 0.0001).

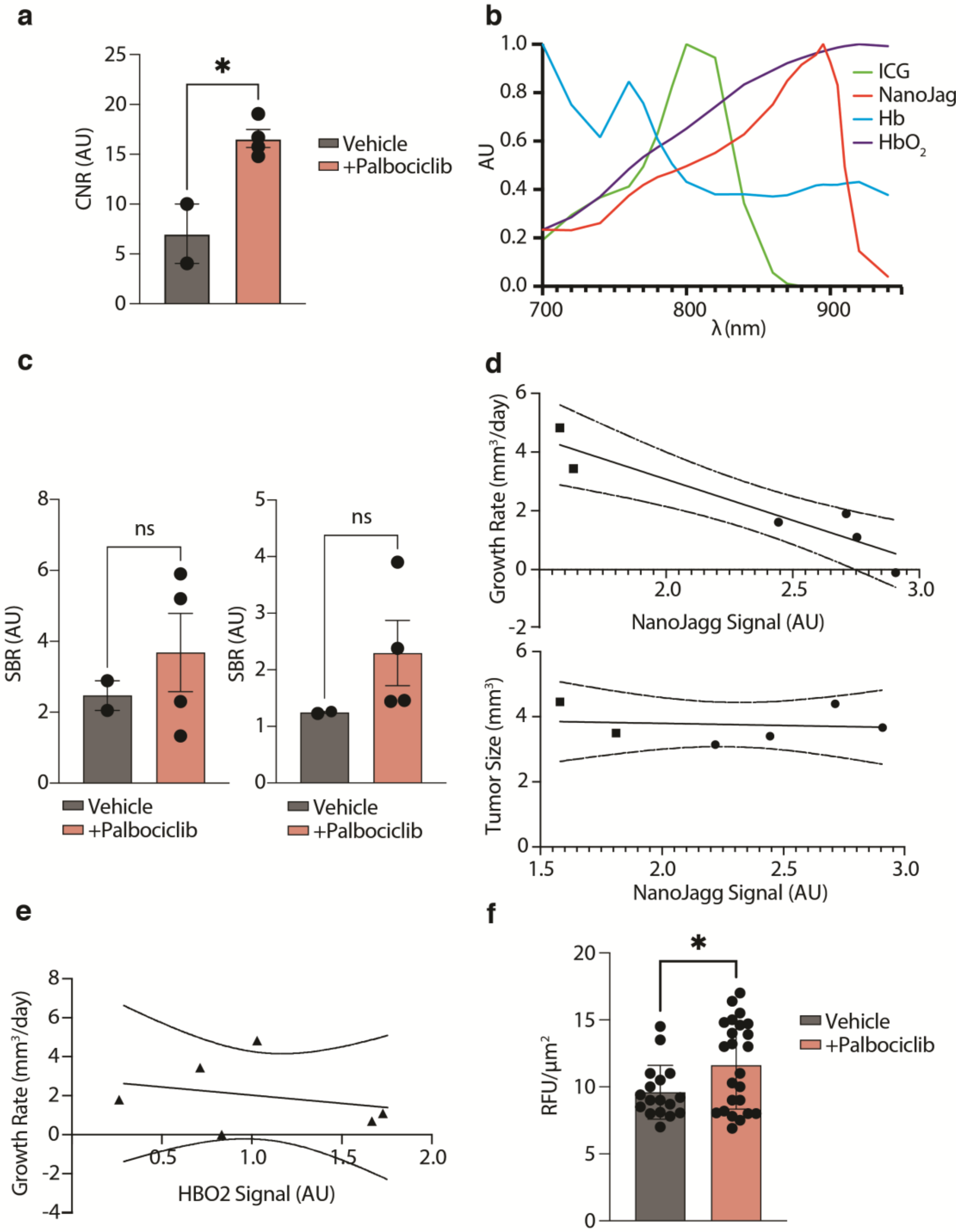

Figure S33. *In vivo* photoacoustic imaging using NanoJaggs. (a) Contrast to noise ratio (CNR) for untreated and treated (senescent) tumors indicating significantly higher CNR for treated tumors (p=0.0138). (b) Absorption spectra used for spectral unmixing employing existing reference spectra of ICG, Hb, and HbO2 and measured spectrum of the NanoJaggs. (c) The signal to background ratio (SBR) of linear spectral unmixed oxygenated hemoglobin (HbO2) and deoxygenated hemoglobin (Hb) are not significantly increased in treated tumors ($p = 0.5099$ and $p = 0.2926$, respectively). (d) NanoJagg signal does not correlate with tumor size ($r^2 = 0.015$, $p = 0.817$, ns), but correlates with the tumor growth rate ($r^2 = 0.875$, $p = 0.0061$, **). (e) HBO2 signal does not correlate with tumor growth rate (r^2=0.112, p=0.516, ns). (f) Quantification of fluorescence per cell in untreated and treated tumors indicating higher fluorescence signal in each investigated cell of the treated tumors (p=0.0301). Data represent mean ± SD, and a Two tailed t test was used to calculate the significance (*$p < 0.05$, **$p < 0.01$, ***$p < 0.001$).

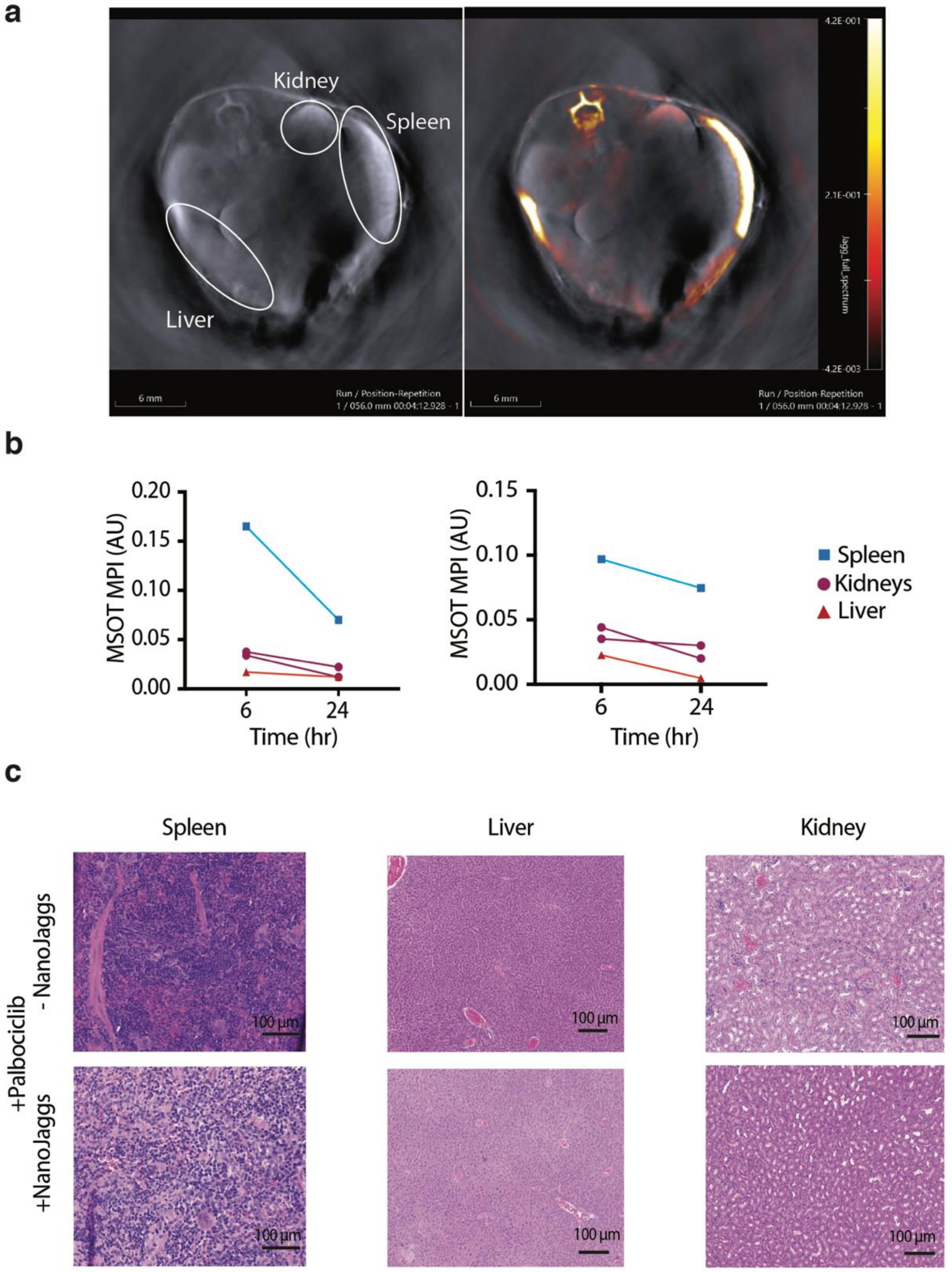

Figure S34. Evaluation of NanoJagg elimination and acute toxicity. (a) Spleen, liver and kidneys are involved in elimination of NanoJaggs as indicated by higher fluorescence in these organs. (b) NanoJagg signal in spleen, liver and kidneys drops significantly after 24 h in two studied mice. (c) H&E staining of spleen, liver and kidney in presence and absence of NanoJagg. NanoJagg treated organs do not appear structurally different indicating there is no acute toxicity for the length of the treatment.

6. Author Contributions

Conceptualization: AGB, DME, LF
Methodology: AGB, DME, LF, ELB, JJ
Investigation: AGB, HO, MH, EG, DM, DME, CS, JG, SM, MD, ELB, MG, ABP, TRE, AV, HFG
Image Analysis: AGB, JJ
Supervision: DME, LF, SEB
Writing: AGB, DME, LF
Review & Editing: AGB, ABP, DME, LF, SEB, ELB, HO, MH, EG, DM, CS, MG, TRE, AV, HFG, JG, SM, DM
